# Conformational bias in SARS-CoV-2 Spike CD4+ T-cell epitope dominance

**DOI:** 10.3389/fimmu.2026.1857103

**Published:** 2026-07-20

**Authors:** Samuel J. Landry, N. Kalaya Steede, Yali Tiomkin, Haley Smith, Ramgopal R. Mettu, Loren Gragert, Judith H. Aberle, Kevin J. Zwezdaryk, Crystal Zheng, Jay K. Kolls, Bronwyn M. Gunn, Amelie E. Murrell, Ivy V. Trinh, John S. Schieffelin, James E. Robinson, Elizabeth B. Norton

**Affiliations:** 1 Department of Biochemistry and Molecular Biology, Tulane University School of Medicine, New Orleans, LA, United States; 2 Department of Computer Science, Tulane University, New Orleans, Louisiana, United States, Tulane University School of Medicine, New Orleans, LA, United States; 3 Department of Medicine, Tulane University School Medicine, New Orleans, LA, United States; 4 Center for Virology, Medical University of Vienna, Vienna, Austria; 5 Department of Microbiology and Immunology, Tulane University School of Medicine, New Orleans, LA, United States; 6 Department of Medicine, Tulane University School of Medicine, New Orleans, LA, United States; 7 Department of Pediatrics, Tulane University School of Medicine, New Orleans, LA, United States; 8 Paul G. Allen School of Global Health, Washington State University, Pullman, WA, United States

**Keywords:** antigen processing, fusogenic conformational change, immunological imprinting, innate to adaptive immune communication, original antigenic sin

## Abstract

**Introduction:**

Epitope-specific T cells provide significant long-lived protection afforded by adaptive immunity to SARS-CoV-2 spike. CD4+ T-cell epitope peptides that are generated by non-ATP-dependent antigen-processing proteases bind with modest specificity to MHC class II molecules in the endo-lysosome. Studies document the influence of antigen-presenting cell type, manner of endocytosis, and antigen conformation on the strength of CD4+ T-cell response. Nevertheless, few studies report changes in epitope dominance due to circumstances of antigen exposure, which could shape proteolytic antigen processing in the class-II pathway because conformational domains limit proteolysis or MHCII binding.

**Methods:**

Processing of SARS-CoV-2 spike was modeled using limited proteolysis of soluble spike trimer, and the effect of spike conformation on CD4+ T-cell epitope dominance was analyzed using IL-2 Elispots responding to two nine-peptide pools from conformationally stable and unstable regions of spike.

**Results:**

Protease-sensitive sites coincided with domain boundaries and other conformationally unstable regions, confirming that structure limits proteolysis. The ratio of CD4+ T-cell response to stable and unstable peptide pools in two non-hospitalized human subjects cohorts distinguished whether exposure was by infection or vaccination.

**Discussion:**

Circumstances of exposure to spike, e.g., spike mRNA vaccination or SARS-CoV-2 infection, could influence populations of antigen presenting cells and their levels of activation, resulting in different patterns of spike fragmentation, peptide loading, and T-cell response. Circumstances of exposure also affect spike conformational changes that contribute to distinct dominance patterns. Thus, epitope dominance patterns potentially indicate exposure history, immune imprinting, and potentially the protectiveness of the CD4+ T-cell response.

## Introduction

Seeking to mitigate the toll of COVID-19 disease caused by SARS-CoV-2, researchers continue to examine immune responses to this novel pathogen. As a major arm of the adaptive immune response, CD4+ T cells have attracted substantial attention for their potential in both positive and negative roles (1). CD4+ T cells generally promote antibody class-switch recombination and antibody affinity maturation in germinal centers, which are prominent in upper airway during COVID-19 infection (2). Apart from supporting antibody, CD4+ T cells promote CD8+ T-cell development, attract phagocytic cells, activate innate immune cell defenses, and deliver regulatory signals that mitigate immunopathology, all of which are thought to be relevant to recovery from COVID-19. Most of these roles have the support of direct evidence in animal models, as well as correlative studies in humans (1, 3, 4). Measuring the expansion of SARS-CoV-2-specific CD4+ T cells is challenged by heterogeneous cellular phenotypes and disparate tissue homing that depend on history and genetics of the host (1, 5). Weak or inhibited triggering of type-I interferon may be an important determinant of disease severity, but immune cell networks that link signaling pathways to tissue damage remain poorly defined (6, 7). Vaccination with the viral spike protein has provided a rapid and strikingly effective protection against severe disease, even though neutralizing antibody faded quickly, potentially due to the lack of long-lived plasma cells in the bone marrow (8). More durable protection may continue to be provided by CD4+ and CD8+ T cells (9–17). Animal models have provided crucial information, but the diversity of human immune systems and the variable history of exposure to viruses, vaccines, and other cross-reactive entities potentially hinders the development of T-cell-based vaccination and therapy (18–20).

CD4+ T-cell epitope dominance refers to the strong or frequent response to a minor fraction of possible epitopes after priming with intact antigen. Peptides generally of 12–20 residues are displayed in class II MHC proteins (MHCII) for recognition by alpha/beta receptors on T cells that also express the CD4 molecule, which binds to a distinct domain of the MHCII (21). Antigen-specific T-cell response is quantified by the frequency of specific T cells, where specificity is established by restimulation of the cells with synthetic peptides that mimic the naturally processed peptides. In a screen for multiple CD4+ T-cell epitopes (hereafter, “CD4+ epitopes”), readout is typically by formation of Elispots by cells that express inflammatory cytokines (e.g., IFNγ or IL-2), or by flow cytometry of cells that express inflammatory cytokines or activation-induced markers (e.g., CD137 and OX40), or by quantitative TCR cloning or sequencing (22). A naturally processed CD4+ epitope typically occurs as a family of overlapping peptides that share a determinant core sequence of 8–9 residues (23). Much of the core is enclosed in the MHCII and is held there with H-bonds to the peptide backbone, and 3–4 peptide sidechains make favorable contacts with shallow pockets in the MHCII groove (24). The T-cell receptor (TCR) binds to the MHCII-peptide complex, typically making contact with 3–4 outward-facing sidechains of the peptide (25). Dominant CD4+ epitopes frequently occur in clusters of overlapping and neighboring sequences that are presented by different allotypes of MHCII or presented by the same MHCII using different binding registers (26–28). For a number of antigens, including HIV envelope glycoprotein and influenza hemagglutinin, this clustering of dominant CD4+ epitopes has been attributed to preferential pathways of proteolytic antigen processing (29–33). These observations led to a mechanistic model and bioinformatic approach to CD4+ epitope prediction based on antigen processing likelihood (APL), wherein the conformationally unstable antigen segments are preferentially cleaved, and then adjacent sequences are bound by MHCII proteins (34, 35).

The complex structure and conformational behavior of the SARS-CoV-2 spike glycoprotein has the potential to modulate its class-II-pathway proteolytic processing and CD4+ epitope dominance. The trimeric SARS-CoV-2 spike glycoprotein is characterized by multiple structural domains connected by domain linkers that articulate conformational states. As a type 1 fusion protein, low pH triggers exposure of a fusion peptide that mediates transfer of the viral genome into the cell cytoplasm (36). For SARS-CoV-2 spike, two proteolytic steps are required. Cleavage of spike (S) into S1 and S2 chains primes the S2 spring-loaded mechanism, and then cleavage at the S2’ site exposes the adjacent fusion peptide (37). Cleavage at the S2’ site is blocked until the receptor-binding domains rotate up to engage the ACE2 receptor on target cells, which acts as a conformational signal mediated through S1 subdomains 1 and 2 (38). These conformational features could influence spike CD4+ epitope dominance. CD4+ epitope dominance in the influenza A hemagglutinin HA1 domain has been attributed to peptide abundance shaped by antigen processing, and the conformational change in HA2 was shown to affect the ability of the protein to restimulate specific T-cells (33).

The rationale for the present study was that COVID-19 severity could be related to CD4+ epitope immunodominance. Severe disease could result from viral pathogenesis in the absence of protective CD4+ T-cell responses or from immunopathology caused by excess or misdirected CD4+ T cell responses. In both scenarios, the immediate cause of differential priming or recall of CD4+ epitope-specific T cells could be variations in the circumstances of antigen exposure, such as antigen dose (39, 40), platform (41), adjuvant (42, 43), age (44), and preexisting cross-reactive immunity (45, 46). The ways in which these circumstances could cause differential CD4+ epitope presentation include variations in antigen conformation, antigen-presenting cell type, and degree of endosomal acidification (47–49). In addition, human leukocyte antigen (HLA) genetics and expression inevitably have interactions with these variations. While a number of studies have reported HLA-allele associations with protection or susceptibility to COVID-19, few have been confirmed in the largest GWAS efforts. Most notably DQB1*06 was associated with vaccine-induced antibody against SARS-CoV-2 spike and reduced breakthrough infection (50). A recent large GWAS study resolved DQB1*06:04, DQA1*01:02, DQA1*01:01, DRB3*01:01, and DPB1*10:01 and found additive effects on antibody and protection (51). These results highlight the CD4+ T-cell helper function to B cells and raise the questions whether HLA polymorphism exerts its effects through CD4+ epitope peptide selectivity and whether some CD4+ epitopes are more protective than others. Nevertheless, studies linking CD4+ epitopes to immunological outcomes often find the CD4+ epitopes to be promiscuously presented by multiple HLA allotypes, a feature that seems to be common to many CD4+ epitopes in SARS-CoV-2 spike (52–55). The role of natural processing of dominant CD4+ epitopes comes to the fore when unravelling mechanisms of immune imprinting and, especially, of “hybrid immune damping” that arises from certain pairings of spike proteins from different viral strains during infection and vaccination (14).

Following early reports of immunodominant CD4+ epitopes, we noted that some of the SARS-CoV-2 spike CD4+ epitopes were located in segments characterized by conformational flexibility, which is unusual for dominant CD4+ epitopes. To probe CD4+ epitope dominance, we designed spike peptide pools that in an IL-2 Elispot assay would discriminate circumstances of priming on the basis of APL score. In the second year of the pandemic, many people became vaccinated, some in their first exposure to SARS-CoV-2 spike and others having previously been infected by the virus. Differences in response to selected CD4+ epitopes following infection *versus* vaccination have previously been noted (18, 46). Here we highlight broad shifts in CD4+ epitope immunodominance in the course of multiple spike exposures and provide evidence that these shifts are based on antigen processing. We also find a similar shift in immunodominance between groups of moderate and mild disease in an unvaccinated, non-hospitalized cohort of infected individuals.

## Results

### Antigen processing likelihood

In order to examine a potential relationship of SARS-CoV-2 spike structure to CD4+ T-cell immunodominance, APL-based CD4+ epitope-prediction profiles for multiple conformations of SARS-CoV-2 spike were compared to CD4+ epitope dominance as reported in the 2023 IEDB database (56). These immunodominant peptides obtained a response from 49-80% of previously infected subjects. The S1 and S2 chains were considered separately because the intact spike polypeptide is cleaved at the S1-S2 junction after shedding or during infection, or cleaved near the S1-S2 junction at an early stage of antigen processing following vaccination (Ref (57) and see below). Multiple S1 and S2 conformations were evaluated for APL, including the following: S1 in the all-RBD-down (PDB:6VXX) and 3-RBD-up (PDB:7DCC) conformations, and S2 in the pre-fusion (PDB:6VXX) or post-fusion (model based on PDB:6B3O) conformations. For S1, APL using any one structure predicted a subset of CD4+ epitopes but did not achieve a significant level of accuracy overall. Notably, scoring of the same overlapping-peptide set by MHCII binding (7-allele method) or by assignment according to observation in immunopeptidomics also did not yield significant accuracy (58, 59) (data not shown). The result with APL is unexpected in view of good accuracy for APL with HIV Env and Influenza HA (60). APL was reasonably accurate with the pre-fusion spike S2 of common cold coronavirus OC43 (and more accurate when combined with MHCII binding), but it was not accurate using the pre-fusion structure of S2 from SARS-CoV-2 (**Supplementary Figure 1**). APL was modestly accurate using the post-fusion conformation of S2 (**Supplementary Figure 2**).

### Limited proteolysis

**Figure 1:**
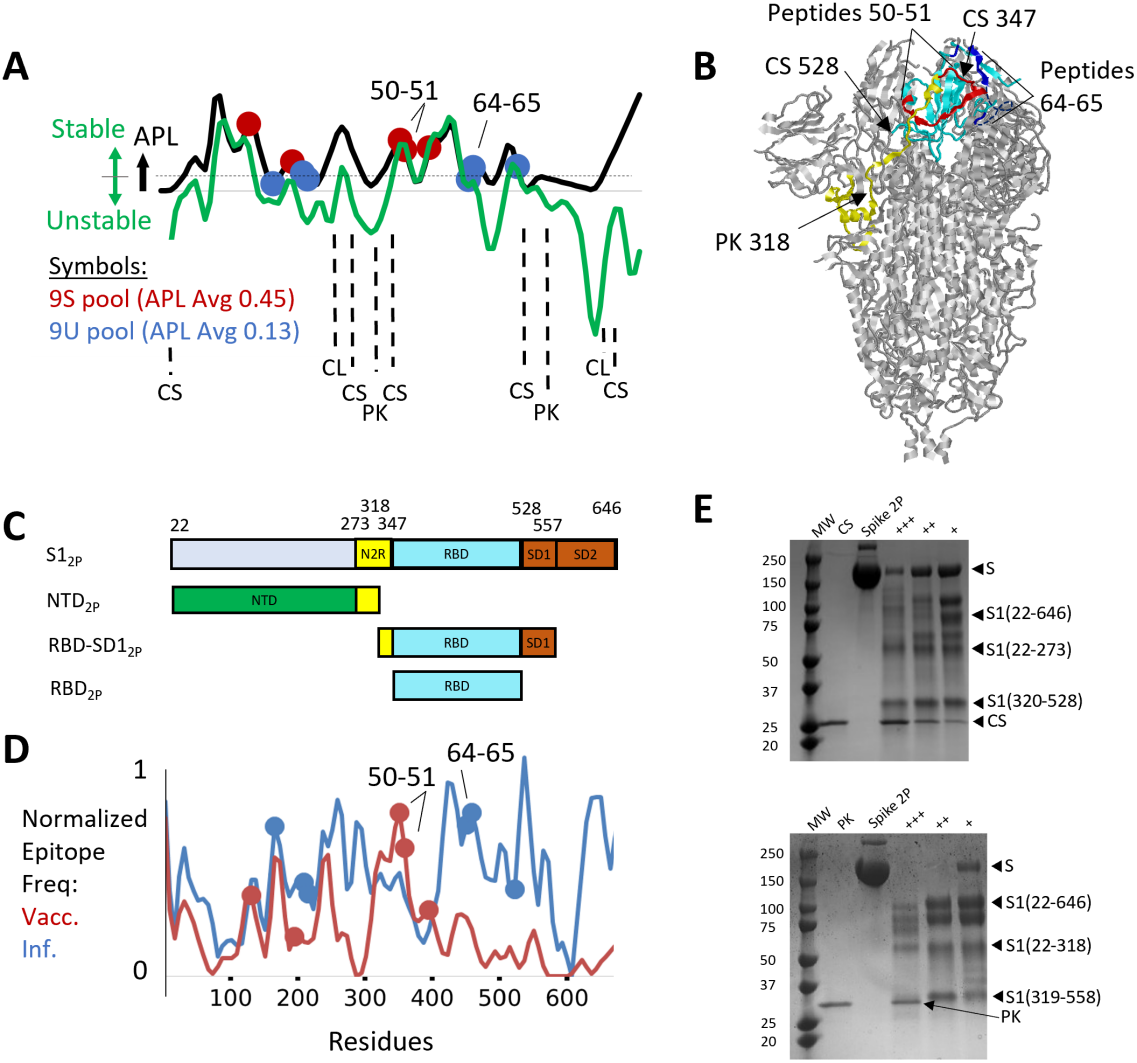
Within the SARS-CoV-2 spike S1 fragment, conformationally unstable, solvent-exposed, protease-sensitive regions more frequently prime CD4+ epitopes during infection than during vaccination. **(A)** APL (black curve) and Aggregate Stability (green curve) for S1 by peptide. The broken horizontal line indicates the average APL for all spike peptides (0.29). Cleavage sites for cathepsin S (CS), proteinase K (PK), and cathepsin L **(CL)** are indicated. **(B)** Ribbon diagram of the intact spike prefusion trimer (PDB: 6VXX), indicating the dominant epitope-containing peptides and flanking protease cleavage sites. **(C)** Diagram of major proteolytic fragments generated by limited proteolysis of spike-2P with CS or PK. **(D)** Profiles of CD4+ epitope dominance generated with mapping data from SARS-CoV-2 infection or vaccination reported in the Immune Epitope Database (IEDB) and normalized by the all-epitope average frequency for the exposure type. Peptides with above- and below-average APL are identified as belonging to the stable and unstable categories (red and blue symbols, respectively). **(E)** Coomassie-stained bands generated by limited proteolysis of recombinant spike-2P with cathepsin S (CS: 1.8, 0.9, or 0.45 µg) or proteinase K (PK: 1.5, 0.06, or 0.03 µg), separated by SDS-PAGE, and identified by tryptic digestion and mass spectrometry. Banding patterns are typical of at least three experiments. The 9U peptides in S1 occur in unstable regions close to protease-sensitive sites (ex., peptides 64-65 at the C-terminus of the RBD); whereas the 9S peptides occur within the NTD and RBD domains (ex., peptides 50-51). IEDB data indicate a dominant response to peptides 64-65 after infection, and a dominant response to peptides 50-51 after vaccination. CL sites were reported by Zhao et al. (Ref. 91).

**Figure 2:**
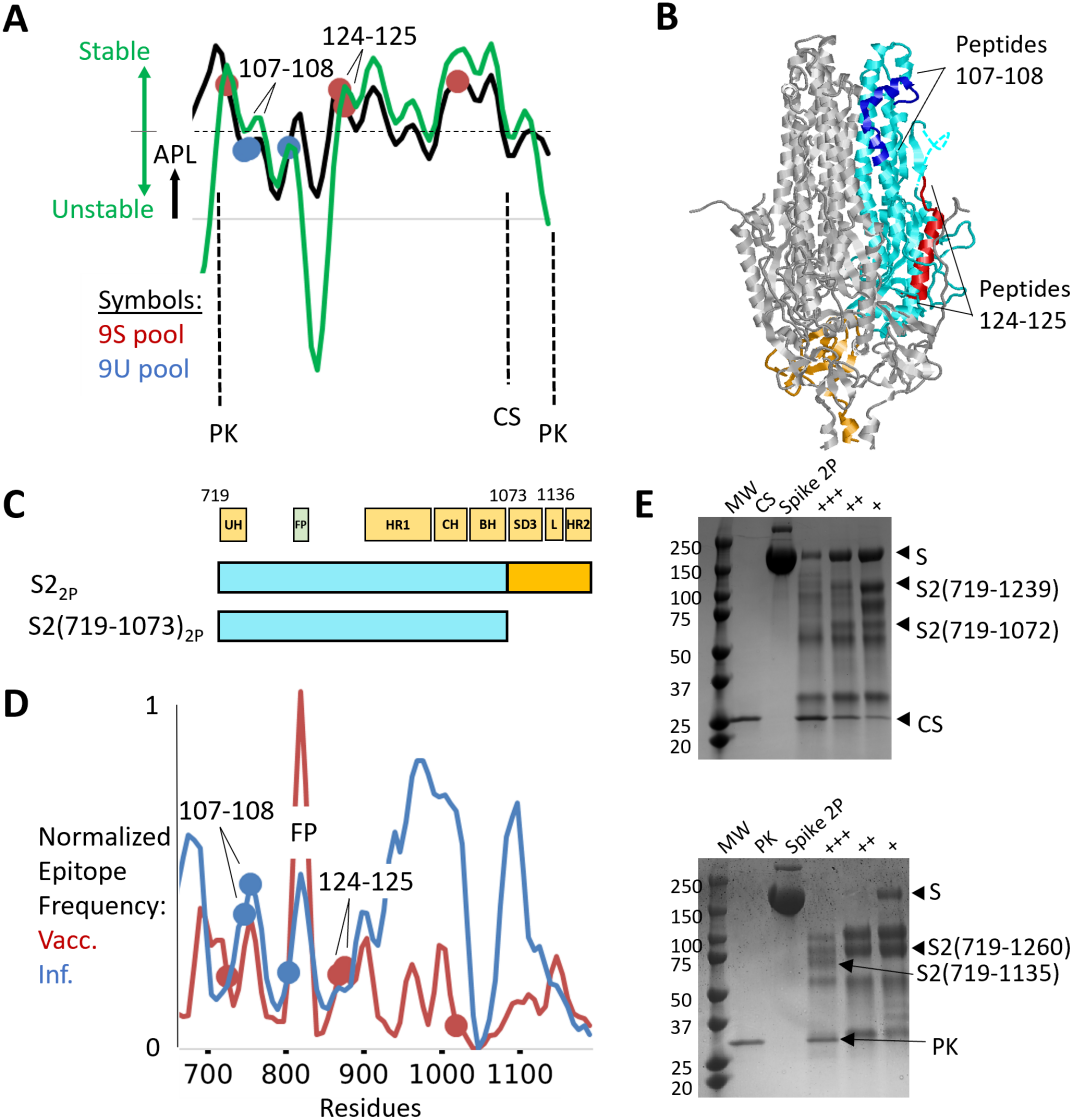
Within the spike S2 fragment, conformationally unstable, solvent-exposed, protease-sensitive regions more frequently prime CD4+ epitopes during infection than during vaccination. **(A)** APL and Aggregate Stability for S2 annotated as in **Figure 1A**. **(B)** Ribbon diagram of the S2 portion of the intact prefusion trimer (PDB: 6VXX). **(C)** Diagram of major proteolytic fragments generated by limited proteolysis of spike-2P with cathepsin S (CS) or proteinase K (PK). Structural domains are as follows (Ref. 66): upstream helix (UH), fusion peptide (FP), heptad repeat 1 (HR1), central helix (CH), beta-hairpin (BH), subdomain 3 (SD3), linker (L), and heptad repeat 2 (HR2). **(D)** Profiles of CD4+ epitope dominance in S2 annotated as in **Figure 1D**. **(E)** Coomassie-stained bands generated by limited proteolysis of recombinant spike-2P with CS (1.8, 0.9, or 0.45 µg) or PK (1.5, 0.06, or 0.03 µg), separated by SDS-PAGE, and identified by tryptic digestion and mass spectrometry (same images as in **Figure 1E**). Banding patterns are representative of at least three experiments.

The modest performance of APL raised the possibility that proteolytic processing of spike was inadequately modeled using the available structures. Limited proteolysis was analyzed for soluble recombinant SARS-CoV-2 spike ectodomains containing proline residues that inhibit conversion to the fusogenic conformation and enhance protein production in cell culture (61, 62). Proteolytic enzymes were selected on the basis of association with class-II-pathway antigen processing (cathepsin S (63)) or of having weak sequence specificity (proteinase K). Large fragments, presumably generated from protease-sensitive sites in the intact spike or in early proteolytic fragments, were extracted from SDS-PAGE gels and subjected to exhaustive tryptic digestion and identification by mass spectrometry. Cleavage sites were mapped with precision limited by the frequency and size-range of non-glycosylated tryptic peptides contained within fragments, which generally place the sites 0–16 residues beyond the terminal tryptic peptide (**Supplementary Tables 1**, **S2**). Several PK- and CS-cleavage sites in spike-2P were mapped to the nearest residue by searching peptide masses with the “no enzyme” setting. The soluble recombinant spike polypeptide expressed as a trimer in mammalian cells is composed of 1274 amino acid residues (including tags) but migrates in SDS-PAGE as approximately 200 kDa due to extensive modification with glycans (64). In addition to proline substitutions (2P or 6P) that stabilize the pre-fusion conformation in S2, residues at the S1-S2 junction have been substituted in order to reduce cleavage by trypsin-like protease activities (61, 62). In spike-6P, the D614G substitution enhances the RBD-up conformation, most likely increasing its sensitivity to cleavage at the S1/S2 site (65). Both cathepsin S (CS) and proteinase K (PK) generated proteolytic fragments of spike-2P or spike-6P that were similar to S1 and S2 fragments (**Figures 1**, **2**; **Supplementary Figures S3**, **S4**, **Supplementary Table 2**). Additional CS- and PK-cleavage sites within S1 and S2 were also evident (**Supplementary Table 1**). In S1 of spike-2P, large fragments were generated by cleavage at several sites in the NTD-to-RBD (N2R) linker and in subdomain 1 (SD1). Both of these protease-sensitive regions occur within conformationally unstable segments, according to the APL-based analysis of the structure (**Figures 1A, C**). In S1 of spike-6P, fragments indicated cleavage at similar sites in the N2R and SD1, but also at an unstable region of the RBD (**Supplementary Figures 3C, D**). In S2 of spike-2P, several large CS and PK fragments resulted from cleavage on the N-terminal flank of the upstream helix and at sites C-terminal to the beta hairpin (66) (**Figures 2A, C**). In S2 of spike-6P, major fragments extended from CS and PK cleavage sites on the C-terminal flanks of the upstream helix and fusion peptide, respectively, to PK sites in the central helix and subdomain 3 (**Supplementary Figures 4 C, D**). Thus, limited proteolysis detects several cleavage-sensitive regions that are common to spike-2P and spike-6P, including the regions of the S1/S2 processing site, N2R, and SD1 and other cleavage sites in spike-6P that were not observed or were shifted in position, compared to in spike-2P. All of the protease-sensitive sites were located in conformationally unstable segments of spike, and utilization of some sites was modulated by proline substitutions that affect spike conformation. Having confirmed that the APL analysis predicts regions of spike proteolytic sensitivity, we addressed the possibility that proteolytic antigen processing varies by individual subject and circumstances of exposure.

### CD4+ T-cell responses to dominant epitopes derived from stable and unstable conformational regions of spike

In order to compare between individuals the frequency of CD4+ T-cell response to stable and unstable regions of spike, 18 peptides were selected from the BEI Resources array on the basis of their dominance in individuals who had recovered from SARS-CoV-2 infection (9, 52). The 18 spike peptides were divided into 9S “stable” and 9U “unstable” pools by the analysis of conformational stability in the “all-RBD-down” cryo-EM structure of the spike trimer (**Supplementary Figure 6**; **Supplementary Table 3**). The respective 9S and 9U peptides have APL values above and below, respectively, the average value (0.29) for the combined 18 peptides. [The average APL for all spike peptides in this structure is 0.25.] None of the selected peptides has more than 50% identity to common cold coronaviruses (CCCV), and none has been reported to recall a T-cell response that was primed by CCCV (**Supplementary Table 3**).

Blood samples were collected as part of the Community Seroepidemiology and Immunity (CSI) study over the period June 12, 2020 to December 5, 2022, roughly spanning delta and omicron BA.1 waves of the pandemic (**Supplementary Table S5**; **Supplementary Figure 7A**). Cryopreserved PBMC samples were tested ex-vivo for IL-2 Elispot formation in response to the 9S and 9U peptide pools. IL-2 was chosen over IFNγ because there would be very little contribution from CD8+ T cells (67), and preliminary studies found equal or greater spot counts when testing for IL-2. PBMCs at 3e5 cells per well were tested in up to six replicates (average, 5 replicates) by stimulation with the peptide pools at 2 µg/mL each peptide. Mutations affecting residues in the 9S-peptide 19 (delta and omicron variants) and in 9U-peptides 30-31, 65, and 108 (omicron variant) potentially affect responses to these peptides (12, 68).

Although the present study initially focused on COVID-19 infection, the rapid deployment of vaccines allowed us to compare immune responses generated by two different pathways of SARS-CoV-2 spike exposure. By June 2021, many subjects had been vaccinated; and thus, we examined the IL-2 responses to 9S and 9U peptide pools for a cohort of 52 subjects that had been infected or that had been infected and then vaccinated with one of the mRNA vaccines (Pfizer or Moderna). For the unvaccinated subjects, the median time since infection was 106 days. For those who were vaccinated, the median time since vaccination (second shot) was 37 days.

**Figure 3:**
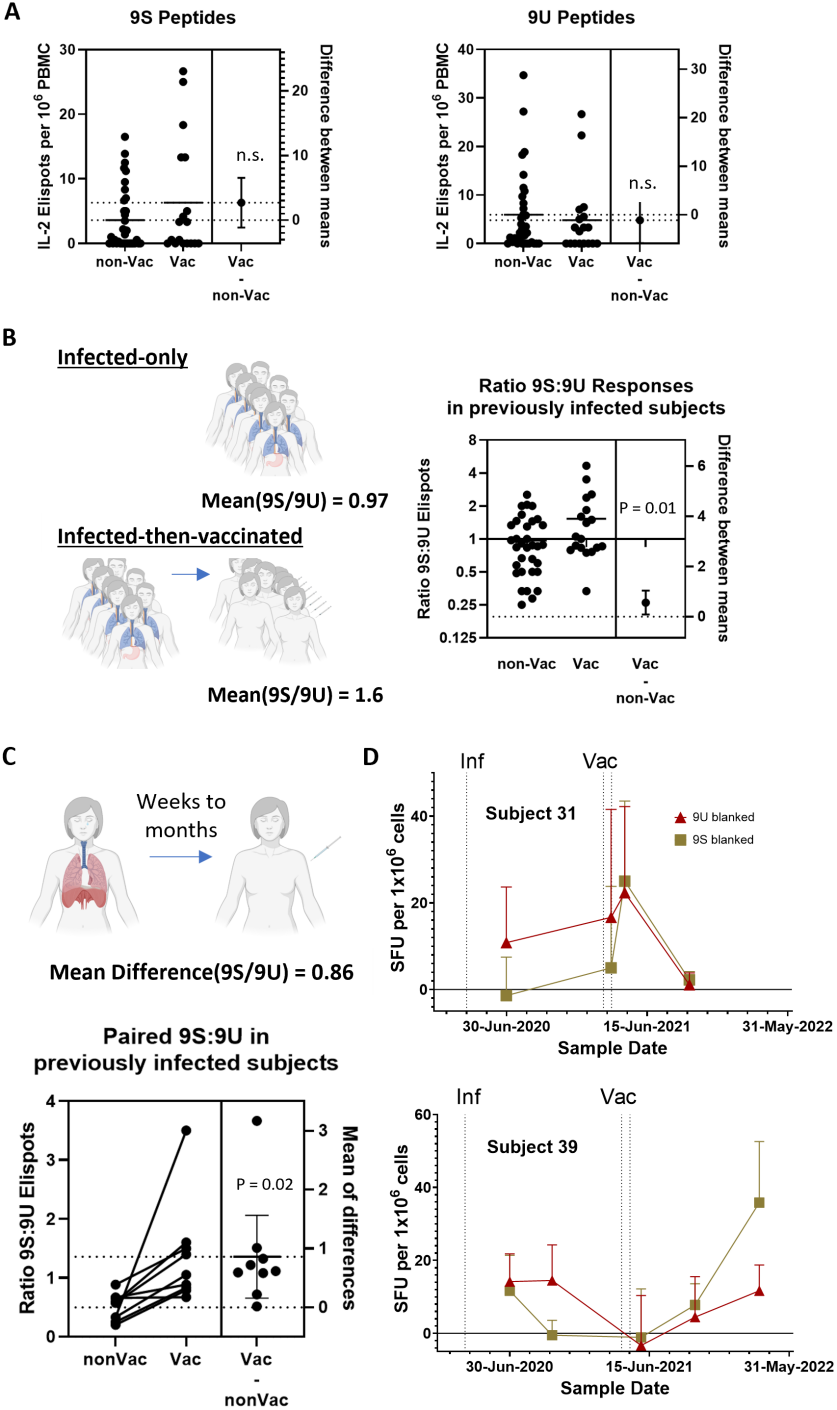
Distinct CD4+ epitope dominance in SARS-CoV-2 spike following infection-alone versus infection plus vaccination. **(A)** Trends toward elevated response to 9S peptides and reduced response to 9U peptides following vaccination. Data corrected for unstimulated signal. N_(non-Vac)_ =35, N_(Vac)_=17, **(B)** Ratio of raw IL-2 Elispot responses to 9S and 9U peptides following infection and vaccination. Values calculated from data in A, prior to correction for unstimulated response. **(C)** Shift in 9S:9U ratio of IL-2 Elispots for individual subjects following their first vaccination (two injections). N = 9. **(D)** Counts of IL-2 Elispots for two subjects before and after vaccination. Vertical lines indicate dates of infection and vaccination. Error bars indicate standard deviation for 3 to 6 replicates wells.

By analysis of blanked IL-2-Elispot counts, obtained by subtraction of the unstimulated counts, the responses to 9S and 9U peptide pools were not significantly different for subjects that had been infected versus infected and vaccinated (**Figure 3A**). In view of the highly variable magnitude of Elispot responses from subject to subject, we also analyzed the 9S:9U ratio of un-blanked Elispot counts, with the expectation that the contribution of non-specific responses would be controlled while allowing more information to emerge from the effective pairing of 9S and 9U signals from each subject. This analysis found a significantly larger 9S:9U ratio in the infected-then-vaccinated subjects, compared to subjects that had been only infected (**Figure 3B**). Paired samples from before and after vaccination also revealed an increase in 9S:9U ratio following vaccination (**Figure 3C**). Longitudinal 9S and 9U responses from two subjects illustrate the selective enhancement of 9S responses upon vaccination (**Figure 3D**).

**Figure 4:**
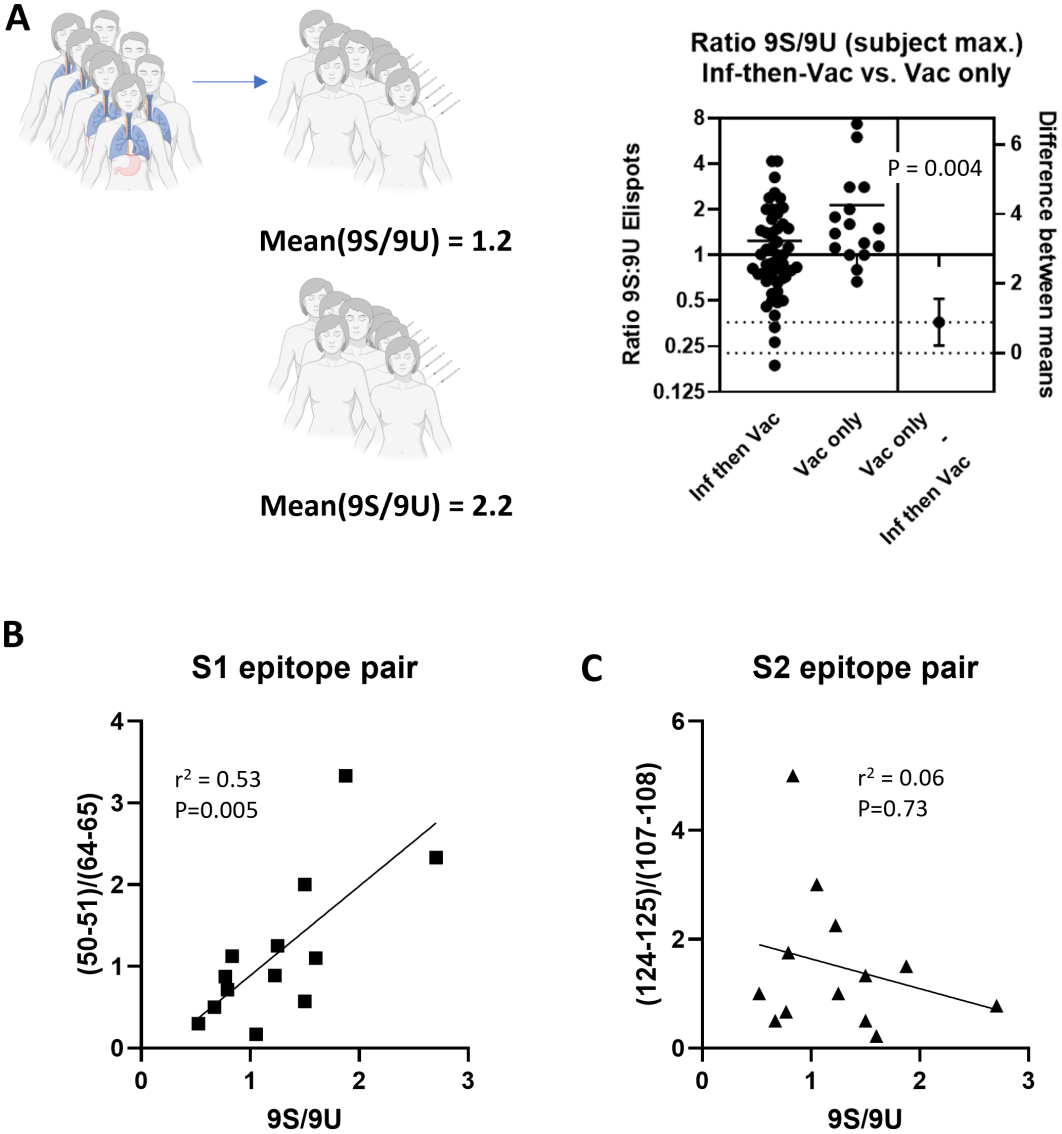
Distinct CD4+ epitope dominance in SARS-CoV-2 spike following infection plus vaccination versus vaccination alone. **(A)** Maximum ratio of raw IL-2 Elispot responses to 9S and 9U pools across all sampling for each subject, where subjects have been infected and then vaccinated or only vaccinated. N(Inf+Vac) = 54, N(Vac) = 17. **(B)** Deconvolution of dominant responses within 9S and 9U pools to S1 peptide pairs by correlation of ratios (50-51):(64-65) and 9S:9U. N = 13. **(C)** No correlation of response for S2 peptide pairs (124-125):(107-108) with that for 9S and 9U pools. N = 13.

Following the continued vaccination of the population, a cohort of 71 subjects was assembled in which 76% had a history of infection and 24% had been vaccinated but never infected (**Supplementary Table S6**; **Supplementary Figure 7B**). Many of the previously infected subjects had a complicated history of spike exposure, e.g., some with two or more infections and some vaccinated plus boosted (3 or more injections). Thus, we identified the maximum 9S:9U ratio for each subject and then compared the mean of maxima for subjects that had been infected and vaccinated with that for subjects that had been only vaccinated. We found that the mean value of maximum 9S:9U ratio for vaccinated-only subjects was significantly higher (**Figure 4A**).

### Partial deconvolution of CD4+ T-cell response to 9S and 9U peptide pools

In order to partially deconvolute the contributions from individual peptides of the 9S and 9U pools, additional IL-2 Elispot assays were conducted on a subset of 13 samples for which the combined 9S and 9U pools had produced >15 spots per 10^6^ PBMC. We reasoned that variation in the ratio of responses for selected peptide pairs, (50-51):(64-65) or (124-125):(107-108), would recapitulate variation in the 9S:9U ratio if these CD4+ epitopes made a substantial contribution to the pooled 9S and 9U responses. Peptides 50–51 of the 9S pool are located in the conformationally stable N-terminal portion of the RBD, and peptides 64–65 of the 9U pool are located in the relatively unstable C-terminal portion of the RBD (**Figure 1**). Peptides 124–125 of the 9S pool are located in a helical region C-terminal to the fusion peptide in the pre-fusion conformation, and peptides 107–108 of the 9U pool are located in the upstream helix (UH) between the S1-S2 cleavage site and S2’ cleavage site (**Figure 2**). Responses to these peptide pairs were expected to be frequent in cohorts because the restricting HLA alleles are common. The combined frequency in the U.S. population of restricting HLA alleles proposed by researchers for each peptide pair exceeds 50% (**Supplementary Table 4**). We found that the (50-51):(64-65) ratio correlated with the 9S:9U ratio. In contrast, the (124-125):(107-108) ratio did not correlate with the 9S:9U ratio (**Figures 4B, C**). Thus, variation in 9S:9U ratio is dominated by the ratio of S1 peptide pairs 50–51 and 64-65.

### CD4+ T-cell responses from an independent cohort of human subjects

**Figure 5:**
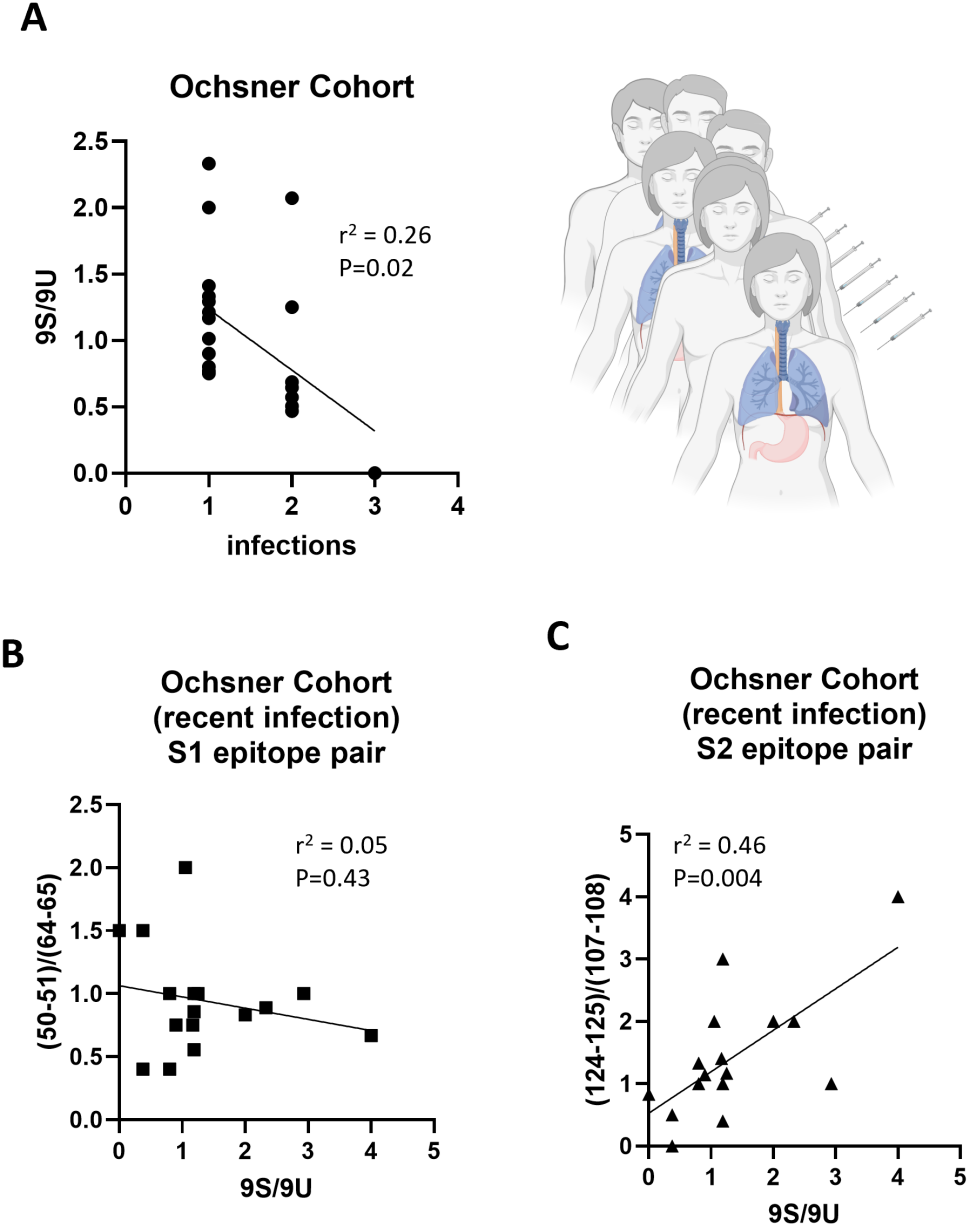
A second human-subjects cohort also reveals an effect of infection on CD4+ epitope dominance in SARS-CoV-2 spike. **(A)** The ratio of raw IL-2 Elispot responses to 9S and 9U pools in a cohort of recently infected subjects diminishes with the number of infections. N = 20. **(B)** Attempted deconvolution of dominant responses finds no correlation of ratios for S1 peptide-pair and peptide-pool responses (50-51):(64-65) and 9S:9U. N = 16. **(C)** Deconvolution of dominant 9S and 9U responses to peptides in S2 by correlation of ratios (124-125):(107-108) and 9S:9U. N = 16.

Seeking corroboration in a separate cohort, we conducted IL-2 Elispot assays with PBMCs from a group of 20 subjects who had recently contracted COVID-19 and sought medical care at Ochsner Health Center (May-August, 2023). Blood samples were collected within 4–14 days after a positive test for COVID-19. Nine of the subjects had been infected once or twice before, and all but one subject had received as few as one and as many as seven injections of mRNA vaccine. To analyze the relationship of 9S:9U ratio to spike exposure, we created a simple scoring system for SARS-CoV-2 spike exposure as the sum of all infections (value 1 for each infection). Evaluation of the linear regression produced a significant negative slope for 9S:9U ratio vs. infection score (**Figure 5A**). Upon partial deconvolution of the 9S and 9U pools in a subset of 16 samples, correlation of peptide pairs with 9S:9U ratio was different from that observed for the CSI cohort. In this case, the (50-51):(64-65) did not correlate with the 9S:9U ratio, but the (124-125):(107-108) ratio did correlate with the 9S:9U ratio (**Figures 5B, C**). Thus, variation in 9S:9U ratio was dominated by the S2 peptide pairs 124–125 and 107–108 in this cohort of subjects.

### Relationship to other cohorts and potentially disease severity

**Figure 6:**
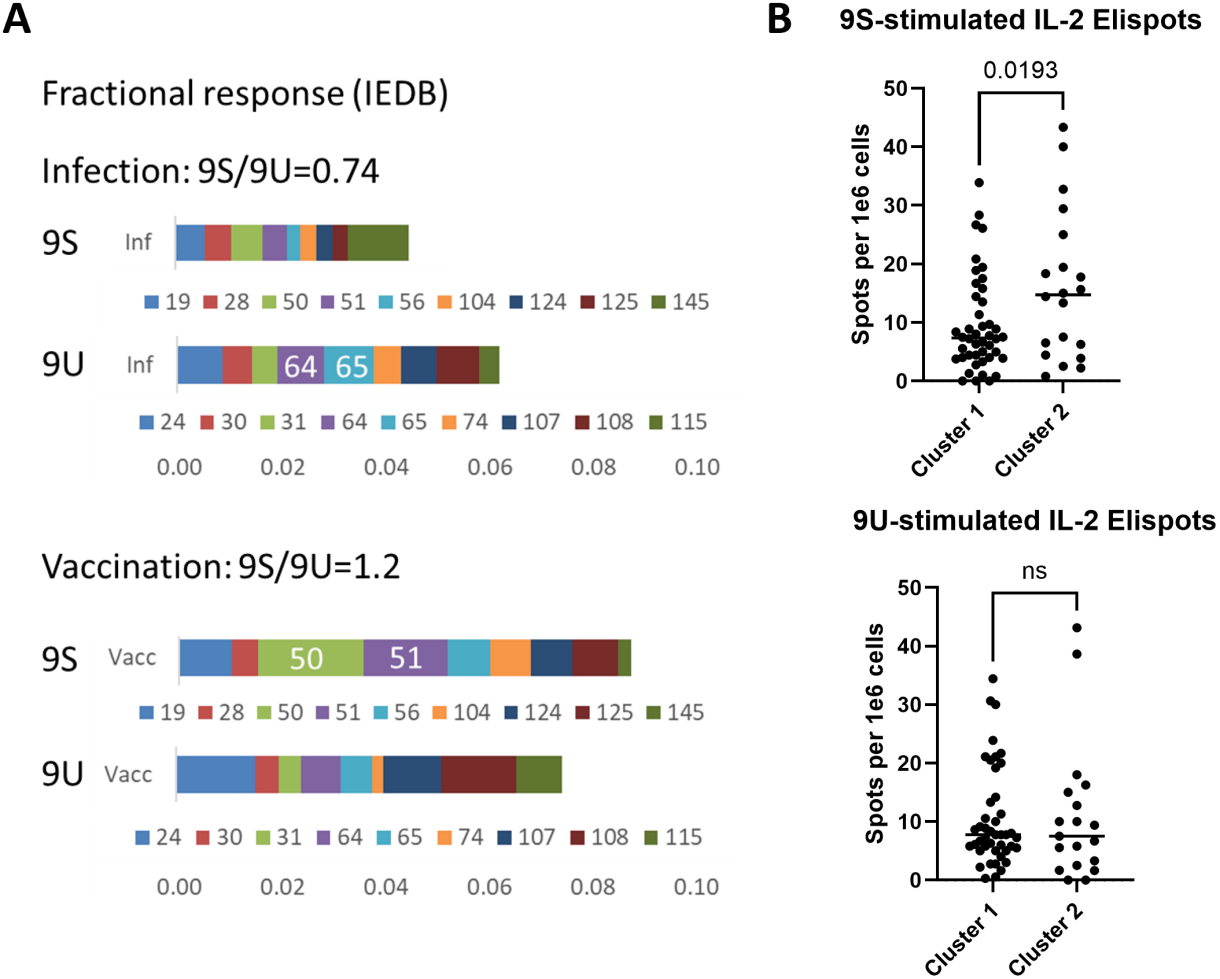
Potential significance of differential response to 9S and 9U peptide pools using IEDB data and clinical phenomena. **(A)** Based on fractional response calculated from epitope frequencies reported in the IEDB: low 9S:9U ratio following infection with SARS-CoV-2 and high 9S:9U ratio following mRNA vaccination with SARS-CoV-2 spike. **(B)** Elevated response to the 9S pool among subjects in cluster 2, who are characterized by Th2-skewed immunity and milder symptoms. Cluster 1, N=44; cluster 2, N=20.

CD4+ epitope profiles from the Immune Epitope Database (IEDB) corroborate the distinct 9S:9U ratios observed for infection and vaccination. CD4+ T-cell responses following infection are more broad, robust, and dominated by different peptide clusters, compared to those following vaccination (**Figures 1D**, **2D**). A particularly noteworthy difference occurs in the region of the RBD. Infection targets 9U peptides 64 and 65 near the C-terminus of the RBD, and vaccination targets 9S peptides 50 and 51 near the N-terminus of the RBD. When the IEDB-reported fractional response for all 9S and 9U peptides was summed, the value of the 9S:9U ratio is less than unity for infection and greater than unity for vaccination (**Figure 6A**).

In a separate effort, a subset of 64 infected-unvaccinated subjects in the present study were partitioned into two clusters on the basis of an array of immune parameters and clinical characteristics. In brief, one cluster reported more severe symptoms and presented Th1-skewed immunity; the other reported milder symptoms and presented less-Th1-skewed immunity (69). From the analysis of IL-2 Elispots, the less-Th1-skewed cluster generated elevated 9S responses in comparison to the Th1-skewed cluster; whereas the 9U responses were indistinguishable for the two clusters (**Figure 6B**).

## Discussion

Distinct patterns of CD4+ T-cell epitope dominance arising from SARS-CoV-2 infection and vaccination indicate that complex differences in immune priming may drive diverse outcomes in disease severity and protection. Here, small pools of peptides representing a few immunodominant CD4+ epitopes from conformationally relatively stable (9S) or unstable (9U) structural contexts in spike report different levels of response, depending on the circumstances of initial exposure. Differences were found in 9S:9U ratio for cross-sections of infected and vaccinated individuals from two different human-subject cohorts and for the longitudinal response of a group of previously infected individuals following vaccination. Additionally, the population-wide CD4+ epitope-mapping data in the IEDB revealed comparable patterns. Together, these studies suggest that the different CD4+ epitope dominance patterns arise from the details of exposure to the spike protein and not from variations in subjects’ HLA-makeup or laboratory experimental approaches. Plausible mechanisms include differences related to vaccine delivery and processing, such as in the tissues of first exposure, local adjuvant effects, cytokine milieu, dendritic cell subsets, and antigen handling in draining lymph nodes (70–72). Although the spike proteins of vaccines have features, such as proline stabilization, that are distinct from the spike proteins of circulating viruses, these modifications are not expected to affect CD4+ T-cell recognition of peptide-MHCII complexes elsewhere in spike. Modifications also would not affect binding to MHCII of distant peptides unless the modifications exerted influence through the intact spike polypeptide or large fragments. Thus, the impact of mechanisms that discriminate priming of CD4+ epitopes within the spike molecule are most likely exerted at the level of antigen processing.

Partial deconvolution of responses to the 9S and 9U peptide pools revealed dominant CD4+ epitope-containing segments in S1 or S2, depending on the human-subjects cohort. In the CSI cohort, the ratio of response (50-51):(64-65) correlated with ratio 9S:9U, but in the Ochsner cohort the ratio (124-125):(107-108) correlated with ratio 9S:9U. At least two distinct features of the cohorts could account for the difference. First, the early-pandemic sampling of the CSI cohort (2020-2022) versus late sampling of the Ochsner cohort (2023) resulted in more cumulative exposure to spike in the Ochsner cohort, and the prevailing SARS-CoV-2 variants will have changed. CD4+ epitope dominance could shift toward S2 because S2 CD4+ epitopes, such as in peptides 124-125, are more conserved across SARS-CoV-2 variants and because the T cells have been maintained at higher levels by crossreaction with CD4+ epitopes from other sources, such as CCCV spike or bacterial antigens. However, significant crossreaction with CCCV appears to be unlikely because the several S2 CD4+ epitopes that were shown to be crossreactive with CCCV spike peptides, including peptides 117, 136, and 141 (46, 54), were deliberately excluded from the 9S and 9U pools. Omicron BA.1 acquired a mutation that affects response to peptide 108, but loss of response to the original peptide would not account for diminishing ratio of response to the S2 peptide pair (124-125:107-108) with increasing number of infections. Responses to peptides 124–125 and 107–108 appear to have been primed by commensal bacteria in significant fractions of the population (45, 73). Thus, changes in response to these peptides could result from changes in resident bacteria. Second, the median interval between exposure and sampling was longer for CSI subjects (one month from vaccination or three months from infection) than for Ochsner subjects (10 days from infection). Since crossreactive T cell responses decay more quickly than novel T cell responses (46), the more-crossreactive S2 responses could still be present at the time of sampling in the Ochsner cohort but already decayed upon sampling in the CSI cohort.

In view of works suggesting that defective CD4+ T-cell responses either allow viral pathogenesis or cause immunopathology (1, 3, 4), we sought to test the hypothesis that CD4+ epitope immunodominance associates with alternate immunological outcomes. Few studies have addressed the question of whether disease severity correlates with the comparative dominance of certain CD4+ epitopes. In an early model for coronavirus infections, C3H/HeJ (I-E^k^) mice experienced severe infections of mouse hepatitis virus (MHV), and C57BL/6 (I-A^b^) mice experienced only mild infections. The authors reported distinct patterns of both CD4+ and CD8+ epitope immunodominance in the response to MHV spike, but pulmonary pathology was associated with only the CD4+ T-cell response, as shaped by the mouse genetic background (74). In humans infected with West Nile virus, distinct CD4+ epitopes have been associated with severe cases, and this has been attributed to a shift in conformation and proteolytic processing of the viral envelope protein E (60, 75). For infections with *Helicobacter pylori*, responses to several DR- and DQ-restricted alleles have been mapped in the protective antigen HpaA; and the response to one DRB1*15:01-restricted CD4+ epitope was associated with less severe gastric disease (76). Although preliminary at best, the present observation of elevated 9S:9U ratio in a cluster of subjects having milder COVID-19 symptoms suggests that epitope dominance in stable spike segments supports better outcomes. Further studies addressing associations of CD4+ epitope dominance with disease severity and vaccine protectiveness are warranted.

Weak processing of dominant CD4+ epitopes in SARS-CoV-2 spike may be associated with disease severity. As noted above, the mean APL calculated for the dominant peptides was not significantly larger than the mean APL for all spike peptides. This result was unexpected because the majority of dominant CD4+ epitopes in a benchmark collection of antigens had above-average APL values (35). The APL algorithm predicts immunodominant CD4+ epitopes in conformationally stable antigen segments, especially those adjacent to unstable, protease-sensitive segments. Only a minority of observed CD4+ epitopes in the benchmark antigens is found to have low APL. These low-APL CD4+ epitopes, which are typically in conformationally unstable segments of 20 or more residues, generally have exceptionally high affinity for the MHCII protein. Such CD4+ epitopes have been dubbed “proteolysis independent” and they may depend on high-affinity MHCII binding for protection from proteolysis (77, 78). CD4+ epitopes in conformationally unstable antigen segments have also been identified for antigens processed *in vitro* under conditions in which the MHCII molecule can bind to the antigen prior to proteolytic processing. In related work, Álvaro-Benito et al. consider antigen-specific cleavage for SARS-CoV-2 and attempt to derive mechanistic insights, while Sadegh-Nasseri and Kim consider the role of structural features that guide binding versus cleavage (79, 80). Given the unusually large fraction of low-APL CD4+ epitopes in spike, we hypothesized that the immunogenicity of these CD4+ epitopes was due to factors that perturb antigen processing.

We sought to bypass the challenges of mapping spike CD4+ epitopes in a cohort of more than fifty human subjects, while discovering meaningful non-MHCII-specific differences in CD4+ epitope immunodominance. The limited size of typical blood samples hinders the analysis of CD4+ epitope dominance, and the problem is exacerbated by the sheer number of possible CD4+ epitopes that can be presented by diverse HLA alleles. Thus, we created rationally designed peptide pools that might reveal immune correlates. In a similar approach, investigators have reported differences in CD4+ T-cell responses to peptide pools spanning S1 and S2 portions of SARS-CoV-2 spike (46, 53, 81, 82). Following COVID-19 infection, the enhanced response to 9U peptides compared to 9S peptides suggests that infection induces the priming of CD4+ epitopes in conformationally unstable antigen segments, which are typically not immunogenic in the dominant antigens of other pathogens. The 9U CD4+ epitopes in S1 occur within unstable regions that are likely to coincide with cleavage sites of early proteolytic fragments generated by antigen processing. Peptides 24 and 64–65 are disordered in cryo-EM structures of the all-RBD-down conformation of the spike trimer (83). Linear antibody epitopes, which must be surface-exposed and able to conform to the antigen-binding site, have been identified in 9U peptides 24 and 30-31, but not in 9S peptides 50-51 (**Figure 1A**) (84). Although not flexibly disordered in the all-RBD-down spike, the local conformation of peptide 74 is reordered during rotation of the RBD upward toward its receptor (83). In a cellular context characterized by aggressive proteolysis, the low conformational stability of 9U CD4+ epitopes should cause these segments to be degraded before they can be loaded into MHCII. How these CD4+ epitopes escape proteolysis is unknown. One possibility is that viral inhibition of innate immunity reduces dendritic cell activation and therefore inhibits proteolytic activity (85–88).

**Figure 7:**
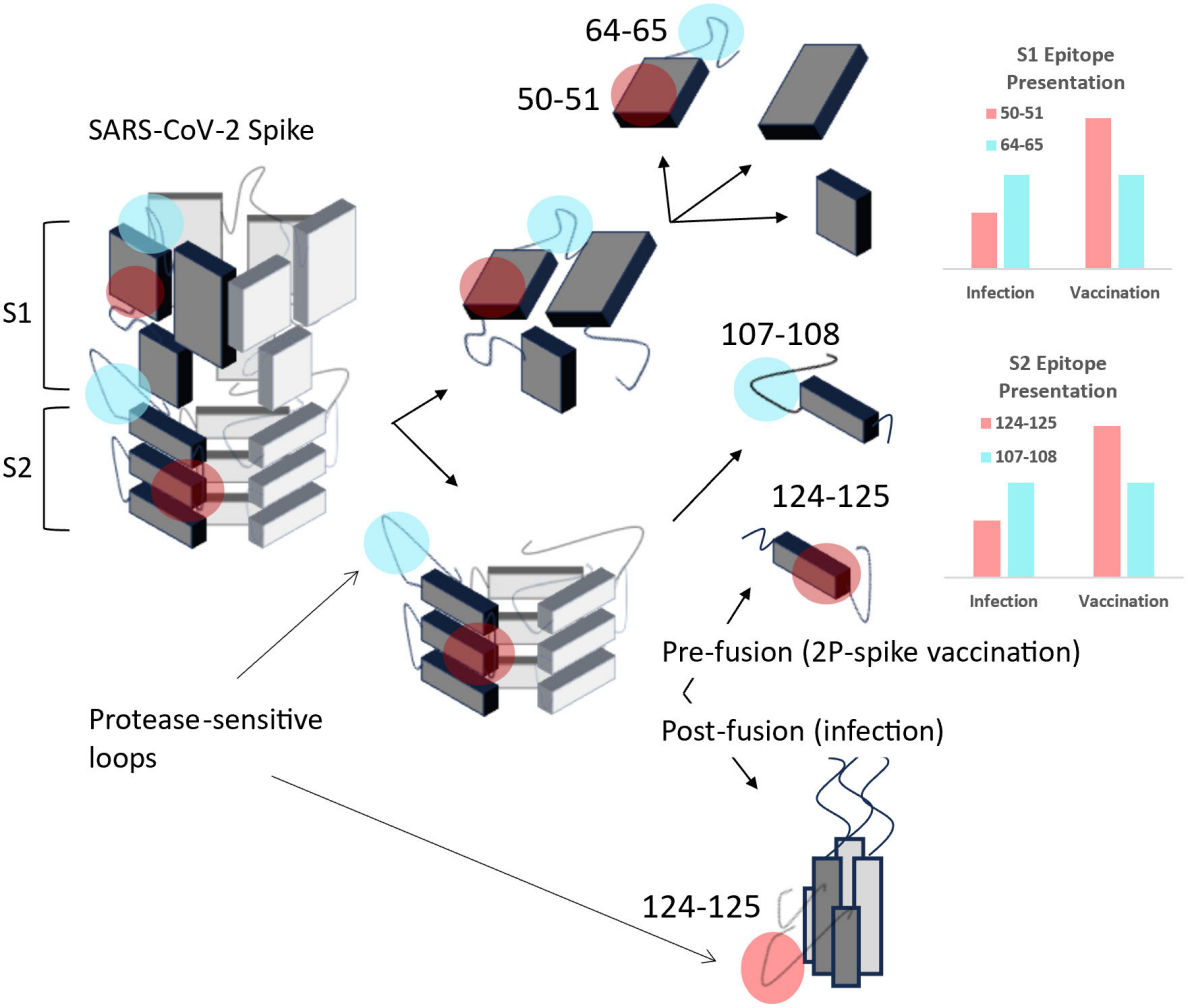
Model for differential processing of CD4+ epitopes based on conformational bias in antigen processing. Proteolysis progressively liberates domains of spike with their associated epitopes. The 9U peptides 64-65 and 107-108 (blue shading) in unstable spike-structure contexts are presented following infection, possibly due to reduced proteolysis resulting from viral suppression of innate immunity and CCCV-induced immune imprinting that limits response to conserved 9S peptides. The 9S peptides 50-51 and 124-125 (red shading) in stable spike-structure contexts are presented following vaccination. Peptides 124-125 may be presented less well from an unstable structural context in the post-fusion conformation.

Explanations for the preferential priming of 9U CD4+ epitopes in COVID-19 infection remain unclear. Tarke et al. point out that that dominant spike CD4+ epitopes have exceptionally broad MHCII affinity (52). Thus, dominance of 9U CD4+ epitopes could be due in part to MHCII-independent factors. The major reorganization of S2 during the post-fusion conformational change very likely affects processing of the S2 CD4+ epitopes. The fusion peptide (peptide 117) converts from being highly immunogenic in vaccinees to modestly immunogenic during infection (**Figure 2D**). As we have previously discussed, this large difference in immunogenicity can be explained by the order-to-disorder transition in the large segment of S2 that spans from the end of the upstream helix (UH) to the beginning of the first heptad repeat (HR1), which potentially exposes the fusion peptide to proteolytic degradation and reduces immunogenicity (**Supplementary Figure 5**) (60). Differences in protease-sensitive sites of spike-2P and spike-6P that are remote from the spike-6P-specific substitutions highlight the potential for conformational changes to affect antigen processing (**Supplementary Table 1**). This mechanism could also explain the higher ratio (124-125):(107-108) in vaccinees because peptides 124–125 are located in the S2 segment that unfolds during infection. It is plausible that the unfolded 124–125 region would be degraded by proteolysis and therefore be less immunogenic during infection (**Figure 7**; **Supplementary Figure S5**). In contrast, peptides 107-108, as part of the UH, would remain folded through the conformational change, remain resistant to proteolysis, and be observed as immunogenic in both types of exposure. In fact, APL predicts increased immunogenicity of peptides 107–108 after the fusogenic conformational change, as is observed for infected subjects.

The shift to greater 9S response in S1 might be explained by an imprinting mechanism. Tarke et al. observed that the dominant SARS-CoV-2 spike CD4+ epitopes generally do not coincide with the dominant CD4+ epitopes of CCCV spikes (certain S2 epitopes notwithstanding (54)) (12). This observation raises the possibility that weak cross-reactivity of CD4+ epitopes reduces their ability to prime naïve T-cell responses, which amounts to a form of antigen imprinting or original antigenic sin. Smith and coworkers have noted that memory responses to the fusion-peptide (peptide-117) are less proliferative than naïve responses to the fusion peptide. Stern and coworkers have also noted that there is no adequate explanation for the absence of a profound amplification of dominant cross-reactive responses to peptide 117 upon infection with SARS-CoV-2 (54). These unresolved issues highlight the fact that a CD4+ epitope’s structural context may control the strength of response. In the context of homologous antigens such as the spikes of SARS-CoV-2 and CCCV, sequence conservation is elevated in the stable regions. Therefore, we might expect a bias against dominant CD4+ epitopes in stable regions, where regular CCCV infections have left their imprint.

Limitations of this study include the small number of proteases used to probe potential processing sites in spike, the use of a soluble, stabilized recombinant spike rather than a native-like membrane-bound spike as a model for infection, and the concentration on initial protease-cleavage sites in the intact spike rather than downstream products of multiple cleavage events. However, multiple studies have documented the extreme and promiscuous sensitivity to proteolytic cleavage of conformationally disordered protein segments, even though different proteases have modest specificity for particular residue types nearest the cleavage site (89, 90). In addition to the CS and PK sites reported here Zhao et al. reported cathepsin L sites in the regions of N2R and SD2 (**Figure 1A**) (91). The disposition of native spike on the viral or cellular membranes is likely to affect its conformation as well as access to proteases, especially for membrane-proximal regions of spike. While this particular consideration was not addressed in this work, the emphasis on local structural environment and how it is modulated by circumstances of exposure is illuminated and should be further studied. The complex pathway of processing and simultaneous loading into MHCII is far from being adequately modeled; nevertheless, three types of CD4+ epitope have been articulated in terms of their structural context, protease-independent, “survivor” (of proteolysis), and “expedited” (as in early intermediate), which have distinct weighting in the APL algorithm (60). The fact that APL is most accurate when taking into account adjacent unstable, protease-sensitive sites suggests that “expedited” processing is an important pathway of MHCII loading. The importance of early processing events has been most elegantly described for the dominant CD4+ epitope of allergen Bet v 1, in which the most immunogenic CD4+ epitope is found in the earliest lysosomal-enzyme digestion products (92). In the present work, individual CD4+ epitopes in SARS-CoV-2 were not scrutinized for immunodominance or processing pathways, and thus it is not possible to identify mechanistic steps. However, the examination of CD4+ epitope regions and subsets of CD4+ epitopes (distinguished by APL scores) supports their comparison in human subjects and with the sparing of limited blood-sample volume.

## Methods

### Human subjects

CSI subjects with suspected or confirmed SARS-CoV-2 infection or who had been vaccinated against SARS-CoV-2 were recruited from the Greater New Orleans community under Tulane Biomedical Institutional Review Board (**Supplementary Table 5** and **6**, federal wide assurance number FWA00002055, under study number 2020-585) (69). Enrolled subjects completed a study questionnaire regarding infection and demographic information and provided a blood sample. A subsample of subjects returned for follow-up visits. Deidentified samples were also utilized from Ochsner Clinic Foundation (FWA Number: FWA00002050) collected from individuals with recent COVID infection (**Supplementary Table 7**). Frozen PBMCs or plasma were utilized from these deidentified samples. Subjects were characterized with respect to prior infections with SARS-CoV-2 and prior SARS-CoV-2-vaccination injections. History of SARS-CoV-2 infection was defined as 1) clear evidence of immunity (SARS-CoV-2 S or N-specific IgG), or 2) detection of plasma viral RNA as described below, or 3) fulfillment of the Centers for Disease Control and Prevention (CDC) case definition of confirmed or probable COVID-19 infection (an individual with a] confirmatory or presumptive laboratory criteria including history of positive SARS-CoV-2 PCR or antigen test or b] absence of negative PCR/antigen test that was performed 2 days prior to 5 days after onset of symptoms and clinical criteria with certain symptoms and fulfill the epidemiological criteria with exposure to a family or household contact with known SARS-CoV-2 (93).

### Antigen processing likelihood

The APL algorithm typically aggregates four sources of information related to local protein conformational flexibility/stability: sequence entropy, crystallographic b-factor, COREX residue stability, and solvent-accessible surface area, which are weighted (0.17, 0.18, 0.51, 0.15, respectively) in accord with a prior optimization for CD4+ epitope prediction in a benchmark set of antigens (35). For SARS-CoV-2 spike, the backbone amide-nitrogen crystallographic b-factors were replaced with reside-by-residue uncertainties in the cryo-EM structure (100-pLDDT). The sequence-entropy profile was generated using 250 homologous proteins discovered in a Blast search with a range of identity (21%-80%) to spike from the Wuhan strain.

### IL-2 Elispot

PVDF plates (Millipore 96-well) were activated as recommended by the manufacturer and washed with 175 µL PBS, pH 7.4. 100 µl of IL-2 monoclonal antibody at 5 µg/ml (clone MQ1-17H12) (Biolegend) was added and incubated at 4 degrees overnight. The next day, plates were washed 4 times with PBS, pH 7.4 then blocked with 175 µl PBS, pH 7.4, 5% BSA at 4 degrees overnight. The next day plates were washed with PBS, pH 7.4 and 150 µl of 2 x 10^6^cells/ml PBMC’s were added to each well. Six replicate wells were composed with no addition (blank wells), 9S, 9U, and nucleocapsid peptide pools at a final concentration of 2 µg/ml. For a single well, ConA was used at final concentration of 4 ug/ml. Plates were incubated for 36–48 hours at 37 degrees C, 5% CO_2_. Plates were then washed 2X with PBS, pH 7.4, and 3X with PBS, pH 7.4, 0.05% Tween-20 as added, and the plate was then 80 µl of 0.0005 mg/ml biotinylated anti-human IL-2 monoclonal antibody (MabTECH) (cloneMT8G10)/PBS, ph7.4/1% BSA/0.05% Tween-20 and left at room temperature for 2 hours. After incubation, plates were washed 3X with PBS, pH 7.4, 0.05% Tween-20; then 80 µL of 1:1000 of Avidin HRP (eBioscience) in PBS was added and left for 1 hour at room temperature in the dark. After incubation plates were washed 4X with PBS, pH 7.4, 0.05% Tween-20, then washed 2X with PBS, pH 7.4; and then developed with 80 µL AEC Substrate solution from the BD Bioscience kit. Development continued for 13 min. Spots were counted using an automated reader system (CTL-ImmunoSpot^®^ S6 Ultimate M2 Analyzer/CTL ImmunoSpot 5.1 Professional DC Software, CTL Europe, Bonn, Germany). For all studies except in **Figure 3A**, raw spot counts for replicate wells were averaged for the individual subject, and then the ratio for peptide pool stimulations (e.g., 9S:9U or 50-51:64-65) were calculated. Mean 9S:9U ratios for groups of subjects (e.g., infected vs. infected-then-vaccinated) were compared by two-tailed t-test in GraphPad Prism, with mean-difference error bars indicating the 95% confidence interval. P-values indicate the probability of the null hypothesis and were considered significant for values below 0.05. For **Figure 3A**, average spot counts for unstimulated wells were subtracted from that of stimulated wells. Negative values were set to zero, and then average spot counts were calculated. For **Figure 4A**, the single highest 9S:9U ratio for each subject was used for comparing groups of subjects. For **Figures 4B, C**, **5B, C**, samples from the cohorts in **Figures 4A**, **5A** were selected for having combined raw 9S + 9U spots greater than 15 per 10^6^ PBMC and then tested for IL-2 Elispots stimulated by peptide pairs. Resulting ratios (e.g., 50-51:64-65) were then tested for correlation (significantly non-zero slope) with 9S:9U ratios from the same subjects using linear regression. **Figures 3**–**5** contain panels created with BioRender.

### Limited proteolysis and peptide mapping

Proteolysis reactions with proteinase K (PK) and cathepsin S were performed in phosphate-citrate buffer at pH 6.6. Each 15 µl reaction mixture contained 15.4 µg 6P protein or 10 ug [microgram] 2P protein and 1.5 µg of PK or cathepsin S. Where indicated, DTT was included in reactions at a final concentration of 5 mM. Reaction mixtures were incubated at 37 °C for 15 min. PK reactions were terminated by the addition of PMSF to 10 mM, and cathepsin S reactions were terminated by addition of 6x SDS protein loading dye, containing 0.1M DTT and heating at 95 °C for 5 minutes. Samples were analyzed by SDS−PAGE using the Bio-Rad TGX gradient gel system. Gels were stained with Coomassie blue and scanned on a BioRad Chemidoc MP imaging system and analyzed with BioRad ImageLab software. Proteolytic cleavage sites were identified by trypsin sequencing of fragments excised from SDS-PAGE gels.

Each gel slice was destained using DI water and vortex. The supernatant was removed and 25mM ammonium bicarbonate and 50% acetonitrile was added. This was repeated until fully destained. Destained gel slices were dehydrated in 100% Optima ACN. The gel was vacuum-dried, and the dried slices were then incubated and vortexed in 25mM DTT in 25mM ammonium bicarbonate for 1 hour. Then 55mM IAA in 25mM ammonium bicarbonate was added and incubated in the dark. The gel was dried with 25mM ammonium bicarbonate in 50% Optima ACN. Additional drying was completed with 100% Optima ACN. The gel was vacuum-dried and resuspended in 25mM ammonium bicarbonate with the addition of mass-spectrometry-grade trypsin incubated at 37 °C overnight. The digestion was stopped using 20% formic acid in water. The solution was vortexed, and the supernatant was transferred to a new LoBinding tube. An additional extraction was performed using 70% Optima ACN in 5% formic acid in water. The extracted supernatant was vacuum-dried, and the peptides were reconstituted in an MS buffer of 2% Optima ACN in 0.1% formic acid centrifuged and transferred into an LC vial.

Each sample was subjected to a 140-minute chromatographic method employing a gradient from 5-25% to acetonitrile in 0.1% formic acid (ACN/FA) over the course of 90 minutes, and 25-95% to acetonitrile in 0.1% formic acid (ACN/FA) over the course of 35 minutes. A gradient to 5% ACN/FA for an additional 15 minutes, a step to 90% ACN/FA for 8 minutes, and a re-equilibration into 2% ACN/FA. Chromatography was carried out in a trap-and-load format using an LC vanquish and autosampler; trap column Pep Map Neo C18 5UM 300µm x 5mm; and the separation column was EASY-Spray Pep Map Neo 1500 bar, 75µm x 500mm. The entire run had a 0.3 µl/min flow rate. Survey scans were performed in the Orbitrap utilizing a resolution of 120, 000 between 375–1500 m/z (customized automated gain control (AGC) target of 300, auto max injection time (IT)). The MS/MS were collected on + 2H to + 7H precursors achieving a standard AGC Data-dependent MS2 scans were performed in the linear ion trap using a HCD Collision Energy of 30%. DDA was performed with one full MS event followed by 41 MS/MS windows in one cycle resulting in a cycle time of 3 s.

Raw data was searched using Proteome Discoverer 3.0 using SEQUEST. The Protein FASTA database was M. musculus (TaxID = 10090) version 2024-11–18 with the SARS-CoV- sequence added. Static modifications included carbamidomethyl/+57.021 Da (C) and dynamic modification of oxidation/+15.995 Da (M). Parent ion tolerance was 10 ppm, fragment mass tolerance was 0.6 Da. For tryptic peptide search, the maximum number of missed cleavages was set to 2. Only high-scoring peptides were considered utilizing a false discovery rate (FDR) of 1%.

## Data Availability

The original contributions presented in the study are included in the article/**Supplementary Material**. Further inquiries can be directed to the corresponding author.
