# Supplementary material for: Conformational bias in SARS-CoV-2 Spike CD4+ T-cell epitope dominance
Source: Front Immunol. 2026 Jul 20;17:1857103. doi: 10.3389/fimmu.2026.1857103 (PMC13429665; doi:10.3389/fimmu.2026.1857103)
Supplement: Supplementary file 1 [file Supplementaryfile1.pdf]

Conformational bias in SARS-CoV-2 Spike CD4+ T-cell epitope dominance

Samuel J. Landry et al.

Supplemental Data

Table S1. Protease cleavage sites in recombinant SARS-CoV-2 spike proteins

	Sites				Notes
**Protease**	**CS**		**PK**		
**Spike**	**2p**	**6P**	**2p**	**6P**	
**S1**					259-260: CatL cleavage (1)
	273±16	273±16			
				311±16	
			318-319		
	347±16			347±16	
				454±16	
		466±16			
	528±16				
			557±16		
		567±16			
					D614G: spike-6P
		635±16			636-637: CatL cleavage (1)
	646±16		646±16	646±16	
					682,683,684,685: GSAS for RRAR in spike-2P and spike-6P
**S2**					
	718-719		718-719		
				766±16	
					815-816: TMPRSS/Cathepsin-S cleavage, dependent on ACE2, blocked in CoV-1 by proline stabilization (2)
					F817P: spike-6P
		836±16			
					A892P: spike-6P
					A899P: spike-6P
					A942P: spike-6P
					K986P, V987P: spike-2P and spike-6P
				1014±16	
	1072-1073				
				1086±16	
			1135-1136		
	1238-1239				
			1260-1261		

Table S2.

Identification of proteolytic fragments using mass spectrometry of tryptic peptides.
Glycan masses estimated to be 2.5 kDa. Mass total compared to MWr estimated from migration in gel relative to MW markers.

CS 2P

**S1**	**22-646**	**Mass (kD)**	**Tryptic peptide**	**P0DTC2**	**MH+ [Da]**	**Theo. MH+ [Da]**
	Chain	70.2	TQLPPAYTNSFTR	22-34	1497.77	1495.754
	11 glycans	27.5	RFDNPVLPFNDGVYFASTEK	78-97	2317.157	2316.13
	total	97.7	RFDNPVLPFNDGVYFASTEKSNIIR	78-102	2902.52	2899.474
	MWr (gel)	87.5	FDNPVLPFNDGVYFASTEK	79-97	2162.056	2160.028
			FDNPVLPFNDGVYFASTEKSNIIR	79-102	2745.406	2743.373
			GWIFGTTLDSK	103-113	1225.642	1224.626
			TQSLLIVNNATNVVIK	114-129	1728.024	1727.006
			VCEFQFCNDPFLGVYYHK	130-147	2325.062	2323.031
			SWMESEFR	151-158	1072.466	1071.456
			SWMESEFR	151-158	1088.46	1087.451
			QGNFKNLR	183-190	977.5397	976.5323
			QGNFKNLREFVFK	183-195	1627.892	1626.875
			EFVFKNIDGYFK	191-202	1507.782	1506.763
			HTPINLVR	207-214	950.565	949.5578
			DLPQGFSALEPLVDLPIGINITR	215-237	2480.389	2478.36
			FQTLLALHR	238-246	1099.65	1098.642
			SYLTPGDSSSGWTAGAAAYYVGYLQPR	247-273	2840.369	2838.337
			YNENGTITDAVDCALDPLSETK	279-300	2428.136	2426.103
			SFTVEKGIYQTSNFR	305-319	1778.906	1776.892
			GIYQTSNFR	311-319	1086.545	1085.537
			FASVYAWNR	347-355	1114.556	1113.548
			RISNCVADYSVLYNSASFSTFK	357-378	2531.243	2529.208
			ISNCVADYSVLYNSASFSTFK	358-378	2374.132	2373.107
			ISNCVADYSVLYNSASFSTFKCYGVSPTK	358-386	3267.552	3265.518
			CYGVSPTK	379-386	912.438	911.4291
			CYGVSPTKLNDLCFTNVYADSFVIR	379-403	2941.437	2939.407
			CYGVSPTKLNDLCFTNVYADSFVIRGDEVR	379-408	3498.714	3495.667
			LNDLCFTNVYADSFVIR	387-403	2048.015	2046.995
			LNDLCFTNVYADSFVIRGDEVR	387-408	2605.284	2603.256
			QIAPGQTGK	409-417	900.5004	899.4945
			QIAPGQTGKIADYNYK	409-424	1767.917	1766.907
			IADYNYK	418-424	887.4378	886.4305
			IADYNYKLPDDFTGCVIAWNSNNLDSK	418-444	3135.496	3133.457
			LPDDFTGCVIAWNSNNLDSK	425-444	2267.064	2266.044
			LPDDFTGCVIAWNSNNLDSKVGGNYNYLYR	425-454	3467.657	3465.617
			VGGNYNYLYR	445-454	1219.598	1218.59
			KSNLKPFER	458-466	1119.64	1118.632
			SNLKPFER	459-466	991.5452	990.5367
			DISTEIYQAGSTPCNGVEGFNCYFPLQSYGFQPTNGVGYQPYR	467-509	4885.222	4882.182
			VVVLSFELLHAPATVCGPK	510-528	2038.142	2037.12
			VVVLSFELLHAPATVCGPKK	510-529	2168.252	2165.215
			KSTNLVK	529-535	790.4896	789.4829
			NKCVNFNFNGLTGTGVLTESNK	536-557	2416.209	2414.177
			NKCVNFNFNGLTGTGVLTESNKK	536-558	2544.28	2542.272
			CVNFNFNGLTGTGVLTESNK	538-557	2173.058	2172.039
			CVNFNFNGLTGTGVLTESNKK	538-558	2302.168	2300.134
			KFLPFQQFGR	558-567	1268.705	1267.695
			FLPFQQFGR	559-567	1140.615	1139.6
			DIADTTDAVR	568-577	1077.53	1076.522
			VYSTGSNVFQTR	635-646	1359.682	1358.67

**22-273**	**Mass (kD)**	**Tryptic peptide**	**P0DTC2**	**MH+ [Da]**	**Theo. MH+ [Da]**
Chain	29.6	TQLPPAYTNSFTR	22-34	1497.768	1495.754
6 glycans	15	RFDNPVLPFNDGVYFASTEK	78-97	2317.152	2316.13
total	44.6	RFDNPVLPFNDGVYFASTEKSNIIR	78-102	2902.52	2899.474
MWr	57.6	FDNPVLPFNDGVYFASTEK	79-97	2162.05	2160.028
		FDNPVLPFNDGVYFASTEKSNIIR	79-102	2745.402	2743.373
		FDNPVLPFNDGVYFASTEKSNIIRGWIFGTTLDSK	79-113	3952.007	3948.981
		SNIIRGWIFGTTLDSK	98-113	1810	1807.97
		GWIFGTTLDSK	103-113	1225.643	1224.626
		TQSLLIVNNATNVVIK	114-129	1728.022	1727.006
		VCEFQFCNDPFLGVYYHK	130-147	2325.06	2323.031
		SWMESEFR	151-158	1088.459	1087.451
		SWMESEFR	151-158	1072.463	1071.456
		EFVFKNIDGYFKIYSK	191-206	2000.068	1998.037
		EFVFKNIDGYFK	191-202	1507.782	1506.763
		HTPINLVR	207-214	950.5656	949.5578
		FQTLLALHR	238-246	1099.649	1098.642
		SYLTPGDSSSGWTAGAAAYYVGYLQPR	247-273	2840.36	2838.337

**347-528**	**Mass (kD)**	**Tryptic peptide**	**P0DTC2**	**MH+ [Da]**	**Theo. MH+ [Da]**
Chain	20.4	FASVYAWNR	347-355	1114.557	1113.548
1 glycan	2.5	RISNCVADYSVLYNSASFSTFK	357-378	2531.228	2529.208
total	22.9	ISNCVADYSVLYNSASFSTFK	358-378	2374.118	2373.107
MWr	31.8	ISNCVADYSVLYNSASFSTFKCYGVSPTK	358-386	3268.543	3265.518
		CYGVSPTK	379-386	912.4363	911.4291
		CYGVSPTKLNDLCFTNVYADSFVIR	379-403	2941.424	2939.407
		LNDLCFTNVYADSFVIR	387-403	2048.004	2046.995
		LNDLCFTNVYADSFVIRGDEVR	387-408	2605.269	2603.256
		GDEVRQIAPGQTGK	404-417	1457.766	1455.755
		QIAPGQTGK	409-417	900.5004	899.4945
		QIAPGQTGKIADYNYK	409-424	1767.915	1766.907
		IADYNYK	418-424	887.4375	886.4305
		IADYNYKLPDDFTGCVIAWNSNNLDSK	418-444	3135.482	3133.457
		LPDDFTGCVIAWNSNNLDSK	425-444	2267.058	2266.044
		LPDDFTGCVIAWNSNNLDSKVGGNYNYLYR	425-454	3467.639	3465.617
		VGGNYNYLYR	445-454	1219.599	1218.59
		KSNLKPFER	458-466	1119.638	1118.632
		SNLKPFER	459-466	991.5444	990.5367
		DISTEIYQAGSTPCNGVEGFNCYFPLQSYGFQPTNGVGYQPYR	467-509	4885.199	4882.182
		VVVLSFELLHAPATVCGPK	510-528	2038.13	2037.12

Identification of proteolytic fragments using mass spectrometry of tryptic peptides.

Glycan masses estimated to be 2.5 kDa. Mass total compared to MWr estimated from migration in gel relative to MW markers.

CS 6P

S1

**22-273**	**Mass (kD)**	**Tryptic peptide**	**P0DTC2**	**MH+ [Da]**	**Theo. MH+ [Da]**
Chain	28.7	TQLPPAYTNSFTR	22-34	1495.754	1495.754
glycans	12.5	RFDNPVLPFNDGVYFASTEKSNIIR	78-102	2899.446	2899.474
total	41.2	FDNPVLPFNDGVYFASTEK	79-97	2316.128	2316.13
MWr (gel)	40.6	GWIFGTTLDSK	103-113	1224.627	1224.626
		SWMESEFR	151-158	1087.452	1087.451
		NIDGYFK	196-202	856.4204	856.4199
		IYSKHTPINLVR	203-214	1440.829	1440.832
		FQTLLALHR	238-246	1098.642	1098.642
		SYLTPGDSSSGWTAGAAAYYVGYLQPR	247-273	2838.338	2838.337

**22-466**	**Mass (kD)**	**Tryptic peptide**	**P0DTC2**	**MH+ [Da]**	**Theo. MH+ [Da]**
Chain	50.6	TQLPPAYTNSFTR	22-34	1495.755	1495.754
glycans	22.5	GVYYPDKVFR	35-44	1243.647	1243.647
total	73.1	RFDNPVLPFNDGVYFASTEK	78-97	2316.13	2316.13
MWr	76.6	FDNPVLPFNDGVYFASTEK	79-97	2160.031	2160.028
		GWIFGTTLDSK	103-113	1224.626	1224.626
		SWMESEFR	151-158	1087.452	1087.451
		NIDGYFK	196-202	856.4202	856.4199
		IYSKHTPINLVR	203-214	1440.832	1440.832
		FQTLLALHR	238-246	1098.642	1098.642
		SYLTPGDSSSGWTAGAAAYYVGYLQPR	247-273	2838.339	2838.337
		SFTVEKGIYQTSNFR	305-319	1776.892	1776.892
		GIYQTSNFR	311-319	1085.538	1085.537
		FASVYAWNR	347-355	1113.546	1113.548
		LNDLCFTNVYADSFVIR	387-403	1989.992	1989.974
		VGGNYNYLYR	445-454	1218.591	1218.59
		KSNLKPFER	458-466	1118.632	1118.632
		SNLKPFER	459-466	990.5367	990.5367

**22-567**	**Mass (kD)**	**Tryptic peptide**	**P0DTC2**	**MH+ [Da]**	**Theo. MH+ [Da]**
Chain	63.7	TQLPPAYTNSFTR	22-34	1495.755	1495.754
glycans	22.5	RFDNPVLPFNDGVYFASTEK	78-97	2316.131	2316.13
total	86.2	FDNPVLPFNDGVYFASTEK	79-97	2160.03	2160.028
MWr	92	GWIFGTTLDSK	103-113	1224.625	1224.626
		SWMESEFR	151-158	1087.451	1087.451
		NIDGYFK	196-202	856.4184	856.4199
		IYSKHTPINLVR	203-214	1440.83	1440.832
		HTPINLVR	207-214	949.5586	949.5578
		DLPQGFSALEPLVDLPIGINITR	215-237	2478.362	2478.36
		FQTLLALHR	238-246	1098.642	1098.642
		SYLTPGDSSSGWTAGAAAYYVGYLQPR	247-273	2838.332	2838.337
		SYLTPGDSSSGWTAGAAAYYVGYLQPRTFLLK	247-278	3440.721	3440.716
		SFTVEKGIYQTSNFR	305-319	1776.89	1776.892
		GIYQTSNFR	311-319	1085.538	1085.537
		FASVYAWNR	347-355	1113.548	1113.548
		LNDLCFTNVYADSFVIR	387-403	1989.99	1989.974
		IADYNYK	418-424	886.4302	886.4305
		VGGNYNYLYR	445-454	1218.59	1218.59
		KSNLKPFER	458-466	1118.632	1118.632
		SNLKPFER	459-466	990.5368	990.5367
		DISTEIYQAGSTPCNGVEGFNCYFPLQSYGFQPTNGVGYQPYR	467-509	4768.098	4768.14
		VVVLSFELLHAPATVCGPK	510-528	1980.1	1980.099
		NKCVNFNFNGLTGTGVLTESNK	536-557	2357.173	2357.155
		KFLPFQQFGR	558-567	1267.692	1267.695
		FLPFQQFGR	559-567	1139.601	1139.6

Identification of proteolytic fragments using mass spectrometry of tryptic peptides.
Glycan masses estimated to be 2.5 kDa. Mass total compared to MWr estimated from migration in gel relative to MW markers.

PK 2P

**S1**	**22-646**	**Mass (kD)**	**Tryptic peptide**	**P0DTC2**	**MH+ [Da]**	**Theo. MH+ [Da]**
	Chain	70.2	TQLPPAYTNSFTR	22-34	1497.769	1495.754
	glycans	27.5	RFDNPVLPFNDGVYFASTEK	78-97	2317.143	2316.130
	total	97.7	RFDNPVLPFNDGVYFASTEKSNIIR	78-102	2901.495	2899.474
	MWr (gel)	110.5	FDNPVLPFNDGVYFASTEK	79-97	2162.046	2160.028
			FDNPVLPFNDGVYFASTEKSNIIRGWIFGTTLDSK	79-113	3952.006	3948.981
			FDNPVLPFNDGVYFASTEKSNIIR	79-102	2745.397	2743.373
			GWIFGTTLDSK	103-113	1225.635	1224.626
			TQSLLIVNNATNVVIK	114-129	1728.014	1727.006
			VCEFQFCNDPFLGVYYHK	130-147	2325.043	2323.031
			NNKSWMESEFR	148-158	1428.645	1427.637
			SWMESEFR	151-158	1072.466	1071.456
			SWMESEFR	151-158	1088.460	1087.451
			EFVFKNIDGYFKIYSK	191-206	2000.056	1998.037
			EFVFKNIDGYFK	191-202	1507.774	1506.763
			NIDGYFKIYSK	196-206	1349.710	1347.694
			IYSKHTPINLVR	203-214	1442.847	1440.832
			DLPQGFSALEPLVDLPIGINITR	215-237	2480.396	2478.360
			FQTLLALHRSYLTPGDSSSGWTAGAAAYYVGYLQPR	238-273	3921.002	3917.961
			FQTLLALHR	238-246	1099.650	1098.642
			SYLTPGDSSSGWTAGAAAYYVGYLQPR	247-273	2840.378	2838.337
			SYLTPGDSSSGWTAGAAAYYVGYLQPRTFLLK	247-278	3442.758	3440.716
			YNENGTITDAVDCALDPLSETK	279-300	2428.124	2426.103
			CTLKSFTVEKGIYQTSNFR	301-319	2281.166	2279.149
			SFTVEKGIYQTSNFR	305-319	1778.907	1776.892
			GIYQTSNFR	311-319	1086.547	1085.537
			VQPTESIVR	320-328	1029.582	1028.573
			FASVYAWNR	347-355	1114.556	1113.548
			RISNCVADYSVLYNSASFSTFK	357-378	2531.226	2529.208
			ISNCVADYSVLYNSASFSTFK	358-378	2374.118	2373.107
			ISNCVADYSVLYNSASFSTFKCYGVSPTK	358-386	3267.526	3265.518
			CYGVSPTKLNDLCFTNVYADSFVIRGDEVR	379-408	3498.701	3495.667
			CYGVSPTK	379-386	912.4383	911.4291
			LNDLCFTNVYADSFVIR	387-403	2049.011	2046.995
			LNDLCFTNVYADSFVIRGDEVR	387-408	2605.277	2603.256
			GDEVRQIAPGQTGK	404-417	1457.771	1455.755
			QIAPGQTGKIADYNYK	409-424	1767.914	1766.907
			QIAPGQTGKIADYNYKLPDDFTGCVIAWNSNNLDSK	409-444	4016.967	4013.934
			QIAPGQTGK	409-417	900.502	899.4945
			IADYNYKLPDDFTGCVIAWNSNNLDSK	418-444	3135.48	3133.457
			IADYNYKLPDDFTGCVIAWNSNNLDSKVGGNYNYLYR	418-454	4336.056	4333.03
			IADYNYK	418-424	887.4383	886.4305
			LPDDFTGCVIAWNSNNLDSKVGGNYNYLYR	425-454	3467.637	3465.617
			LPDDFTGCVIAWNSNNLDSK	425-444	2267.058	2266.044
			VGGNYNYLYR	445-454	1219.598	1218.59
			KSNLKPFER	458-466	1119.642	1118.632
			SNLKPFER	459-466	991.5457	990.5367
			DISTEIYQAGSTPCNGVEGFNCYFPLQSYGFQPTNGVGYQPYR	467-509	4885.231	4882.182
			VVVLSFELLHAPATVCGPK	510-528	2038.122	2037.12
			VVVLSFELLHAPATVCGPKK	510-529	2167.232	2165.215
			KSTNLVK	529-535	790.4905	789.4829
			STNLVKNKCVNFNFNGLTGTGVLTESNK	530-557	3059.572	3056.547
			NKCVNFNFNGLTGTGVLTESNKK	536-558	2544.295	2542.272
			NKCVNFNFNGLTGTGVLTESNK	536-557	2416.167	2414.177
			CVNFNFNGLTGTGVLTESNKK	538-558	2302.151	2300.134
			CVNFNFNGLTGTGVLTESNKKFLPFQQFGR	538-567	3423.746	3420.716
			CVNFNFNGLTGTGVLTESNK	538-557	2173.054	2172.039
			KFLPFQQFGR	558-567	1269.71	1267.695
			FLPFQQFGR	559-567	1140.608	1139.6
			DIADTTDAVR	568-577	1077.53	1076.522
			VYSTGSNVFQTR	635-646	1359.679	1358.67

**22-319**	**Mass (kD)**	**Tryptic peptide**	**P0DTC2**	**MH+ [Da]**	**Theo. MH+ [Da]**
Chain	33.9	TQLPPAYTNSFTR	22-34	1497.769	1495.754
glycans	20	RFDNPVLPFNDGVYFASTEK	78-97	2317.139	2316.13
total	53.9	FDNPVLPFNDGVYFASTEK	79-97	2162.043	2160.028
MWr	58.4	FDNPVLPFNDGVYFASTEKSNIIRGWIFGTTLDSK	79-113	3952.005	3948.981
		FDNPVLPFNDGVYFASTEKSNIIR	79-102	2745.393	2743.373
		SNIIRGWIFGTTLDSK	98-113	1809.986	1807.97
		GWIFGTTLDSK	103-113	1225.634	1224.626
		TQSLLIVNNATNVVIK	114-129	1728.015	1727.006
		VCEFQFCNDPFLGVYYHK	130-147	2325.044	2323.031
		SWMESEFR	151-158	1072.464	1071.456
		SWMESEFR	151-158	1088.459	1087.451
		QGNFKNLR	183-190	977.5402	976.5323
		EFVFKNIDGYFKIYSK	191-206	2000.053	1998.037
		EFVFKNIDGYFK	191-202	1507.771	1506.763
		NIDGYFKIYSK	196-206	1348.701	1347.694
		HTPINLVR	207-214	950.5658	949.5578
		FQTLLALHR	238-246	1099.65	1098.642
		SYLTPGDSSSGWTAGAAAYYVGYLQPR	247-273	2840.352	2838.337
		YNENGTITDAVDCALDPLSETK	279-300	2427.121	2426.103
		GIYQTSNFR	311-319	1086.545	1085.537

**319-557**	**Mass (kD)**	**Tryptic peptide**	**P0DTC2**	**MH+ [Da]**	**Theo. MH+ [Da]**
Chain	26.6	[F].RVQPTESIVR	319-328	1186.69	1184.675
glycans	5	VQPTESIVR	320-328	1029.581	1028.573
total	31.6	FASVYAWNR	347-355	1114.561	1113.548
MWr	34	RISNCVADYSVLYNSASFSTFK	357-378	2530.23	2529.208
		ISNCVADYSVLYNSASFSTFK	358-378	2374.125	2373.107
		ISNCVADYSVLYNSASFSTFKCYGVSPTK	358-386	3268.551	3265.518
		CYGVSPTK	379-386	912.4351	911.4291
		CYGVSPTKLNDLCFTNVYADSFVIR	379-403	2941.439	2939.407
		LNDLCFTNVYADSFVIR	387-403	2049.028	2046.995
		LNDLCFTNVYADSFVIRGDEVR	387-408	2605.296	2603.256
		GDEVRQIAPGQTGK	404-417	1457.766	1455.755
		QIAPGQTGK	409-417	900.4998	899.4945
		QIAPGQTGKIADYNYK	409-424	1767.917	1766.907
		QIAPGQTGKIADYNYKLPDDFTGCVIAWNSNNLDSK	409-444	4016.973	4013.934
		IADYNYK	418-424	887.4369	886.4305
		IADYNYKLPDDFTGCVIAWNSNNLDSKVGGNYNYLYR	418-454	4336.083	4333.03
		IADYNYKLPDDFTGCVIAWNSNNLDSK	418-444	3135.485	3133.457
		LPDDFTGCVIAWNSNNLDSK	425-444	2267.059	2266.044
		LPDDFTGCVIAWNSNNLDSKVGGNYNYLYR	425-454	3467.653	3465.617
		VGGNYNYLYR	445-454	1219.602	1218.59
		KSNLKPFER	458-466	1119.636	1118.632
		SNLKPFER	459-466	991.5437	990.5367
		DISTEIYQAGSTPCNGVEGFNCYFPLQSYGFQPTNGVGYQPY[illegible]	467-509	4885.209	4882.182
		VVVLSFELLHAPATVCGPK	510-528	2038.132	2037.12
		KSTNLVK	529-535	790.4888	789.4829
		STNLVKNK	530-537	904.5321	903.5258
		CVNFNFNGLTGTGVLTESNK	538-557	2173.055	2172.039

Identification of proteolytic fragments using mass spectrometry of tryptic peptides.

Glycan masses estimated to be 2.5 kDa. Mass total compared to MWr estimated from migration in gel relative to MW markers.

PK 6P

**S1**	**22-646**	**Mass (kD)**	**Tryptic peptide**	**P0DTC2**	**MH+ [Da]**	**Theo. MH+ [Da]**
	Chain	68.8	TQLPPAYTNSFTR	22-34	1495.755	1495.754
	glycans	27.5	RFDNPVLPFNDGVYFASTEK	78-97	2316.130	2316.130
	total	96.3	FDNPVLPFNDGVYFASTEK	79-97	2160.027	2160.028
	MWr (gel)	97.6	GWIFGTTLDSK	103-113	1224.626	1224.626
			SWMESEFR (oxidized)	151-169	544.229	1087.451
			NIDGYFK	196-202	856.420	856.420
			HTPINLVR	207-214	949.559	949.558
			FQTLLALHR	238-246	1098.643	1098.642
			SYLTPGDSSSGWTAGAAAYYVGYLQPR	247-273	2838.338	2838.337
			GIYQTSNFR	311-319	1085.538	1085.537
			FASVYAWNR	347-355	1113.548	1113.548
			IADYNYK	418-424	886.431	886.431
			VGGNYNYLYR	445-454	1218.591	1218.590
			KSNLKPFER	458-466	1118.632	1118.632
			SNLKPFER	459-466	990.537	990.537
			VVVLSFELLHAPATVCGPK	510-528	1980.103	1980.099
			KFLPFQQFGR	558-567	1267.696	1267.695
			FLPFQQFGR	559-567	1139.601	1139.600
			DIADTTDAVR	568-577	1076.522	1076.522
			VYSTGSNVFQTR	635-646	1358.669	1358.6699

**311-646**	**Mass (kD)**	**Tryptic peptide**	**P0DTC2**	**MH+ [Da]**	**Theo. MH+ [Da]**
Chain	38.03	GIYQTSNFR	311-319	1085.538	1085.537
glycans	10	VQPTESIVR	320-328	1028.575	1028.573
total	48.03	FASVYAWNR	347-355	1113.548	1113.548
MWr	56.5	ISNCVADYSVLYNSASFSTFK	358-378	2316.089	2316.085
		LNDLCFTNVYADSFVIR	387-403	1989.975	1989.974
		QIAPGQTGKIADYNYK	409-424	1766.907	1766.907
		IADYNYK	418-424	886.4296	886.4305
		LPDDFTGCVIAWNSNNLDSK	425-444	2209.017	2209.023
		VGGNYNYLYR	445-454	1218.59	1218.59
		KSNLKPFER	458-466	1118.633	1118.632
		SNLKPFER	459-466	990.5374	990.5367
		DISTEIYQAGSTPCNGVEGFNCYFPLQSYGFQPTNGVGYQPYI	467-509	4768.101	4768.14
		VVVLSFELLHAPATVCGPK	510-528	1980.097	1980.099
		KFLPFQQFGR	558-567	1267.695	1267.695
		FLPFQQFGR	559-567	1139.599	1139.6
		DIADTTDAVR	568-577	1076.521	1076.522
		VYSTGSNVFQTR	635-646	1358.67	1358.67

**347-454**	**Mass (kD)**	**Tryptic peptide**	**P0DTC2**	**MH+ [Da]**	**Theo. MH+ [Da]**
Chain	12.2	FASVYAWNR	347-355	1113.551	1113.548
glycans	0	IADYNYK	418-424	886.4307	886.4305
total	12.2	VGGNYNYLYR	445-454	1218.59	1218.59
MWr	11.6				

Identification of proteolytic fragments using mass spectrometry of tryptic peptides.

Glycan masses estimated to be 2.5 kDa. Mass total compared to MWr estimated from migration in gel relative to MW markers.

CS 2P

**S2**	**719-1239**	**Mass (kD)**	**Tryptic peptide**	**P0DTC2**	**MH+ [Da]**	**Theo. MH+ [Da]**
	Chain	57.2	[F].TISVTTEILPVSMTK	719-733	1636.893	1635.887
	glycans	16	TSVDCTMYICGDSTECSNLLLQYGSFCTQLNR	734-765	3795.646	3793.627
	total	73.2	ALTGIAVEQDK	766-776	1145.629	1144.621
	MWr (gel)	116.4	ALTGIAVEQDKNTQEVFAQVK	766-786	2291.237	2289.209
			NTQEVFAQVK	777-786	1164.613	1163.606
			NTQEVFAQVKQIYK	777-790	1696.924	1695.906
			DFGGFNFSQILPDPSKPSK	796-814	2082.051	2081.034
			RSFIEDLLFNK	815-825	1383.768	1381.747
			SFIEDLLFNK	816-825	1226.661	1225.646
			SFIEDLLFNKVTLADAGFIK	816-835	2243.252	2241.217
			VTLADAGFIK	826-835	1035.603	1034.588
			VTLADAGFIKQYGDCLGDIAAR	826-847	2356.209	2354.181
			QYGDCLGDIAAR	836-847	1339.623	1338.611
			FNGIGVTQNVLYENQK	906-921	1825.957	1823.929
			FNGIGVTQNVLYENQKLIANQFNSAIGK	906-933	3082.646	3080.616
			LIANQFNSAIGK	922-933	1276.719	1275.706
			LIANQFNSAIGKIQDSLSSTASALGK	922-947	2636.438	2634.41
			IQDSLSSTASALGK	934-947	1378.733	1377.722
			IQDSLSSTASALGKLQDVVNQNAQALNTLVK	934-964	3228.751	3226.728
			LQDVVNQNAQALNTLVK	948-964	1869.047	1868.024
			QLSSNFGAISSVLNDILSR	965-983	2023.096	2021.066
			LDPPEAEVQIDR	984-995	1383.717	1381.696
			LQSLQTYVTQQLIR	1001-1014	1692.975	1690.949
			ASANLAATK	1020-1028	847.4778	846.468
			ASANLAATKMSECVLGQSK	1020-1038	1966.988	1965.973
			ASANLAATKMSECVLGQSK	1020-1038	1983.986	1981.968
			MSECVLGQSK	1029-1038	1139.532	1138.523
			MSECVLGQSK	1029-1038	1155.529	1154.518
			RVDFCGK	1039-1045	882.4391	881.4298
			GYHLMSFPQSAPHGVVFLHVTYVPAQEK	1046-1073	3142.628	3139.582
			GYHLMSFPQSAPHGVVFLHVTYVPAQEK	1046-1073	3158.623	3155.577
			NFTTAPAICHDGK	1074-1086	1432.677	1431.669
			EGVFVSNGTHWFVTQR	1092-1107	1865.94	1863.914
			NHTSPDVDLGDISGINASVVNIQK	1158-1181	2495.293	2493.258
			EIDRLNEVAK	1182-1191	1187.652	1186.643
			NLNESLIDLQELGK	1192-1205	1586.86	1585.843
			YEQGSGYIPEAPR	1206-1218	1467.702	1466.691
			DGQAYVR	1219-1225	809.4045	808.3948
			DGQAYVRKDGEWVLLSTFLGR	1219-1239	2412.277	2410.251
			KDGEWVLLSTFLGR	1226-1239	1622.898	1620.874
			DGEWVLLSTFLGR	1227-1239	1493.801	1492.779

**719-1073**	**Mass (kD)**	**Tryptic peptide**	**P0DTC2**	**MH+ [Da]**	**Theo. MH+ [Da]**
Chain	38.6	[F].TISVTTEILPVSMTK		1636.895	1635.887
glycans	2	TSVDCTMYICGDSTECSNLLLQYGSFCTQLNR	734-765	3795.65	3793.627
total	40.6	ALTGIAVEQDK	766-776	1145.63	1144.621
MWr	57.6	ALTGIAVEQDKNTQEVFAQVK	766-786	2290.231	2289.209
		ALTGIAVEQDKNTQEVFAQVKQIYK	766-790	2824.553	2821.509
		NTQEVFAQVK	777-786	1164.613	1163.606
		NTQEVFAQVKQIYK	777-790	1696.923	1695.906
		DFGGFNFSQILPDPSKPSK	796-814	2083.063	2081.034
		RSFIEDLLFNK	815-825	1383.772	1381.747
		SFIEDLLFNK	816-825	1226.657	1225.646
		SFIEDLLFNKVTLADAGFIK	816-835	2243.23	2241.217
		SFIEDLLFNKVTLADAGFIKQYGDCLGDIAAR	816-847	3563.831	3560.809
		VTLADAGFIK	826-835	1035.602	1034.588
		VTLADAGFIKQYGDCLGDIAAR	826-847	2356.211	2354.181
		QYGDCLGDIAAR	836-847	1339.624	1338.611
		DLICAQK	848-854	848.4424	847.4342
		FNGIGVTQNVLYENQK	906-921	1824.947	1823.929
		FNGIGVTQNVLYENQKLIANQFNSAIGKIQDSLSSTASALGK	906-947	4442.344	4439.321
		LIANQFNSAIGK	922-933	1276.718	1275.706
		LIANQFNSAIGKIQDSLSSTASALGK	922-947	2636.438	2634.41
		IQDSLSSTASALGK	934-947	1378.733	1377.722
		IQDSLSSTASALGKLQDVVNQNAQALNTLVK	934-964	3228.749	3226.728
		LQDVVNQNAQALNTLVK	948-964	1870.048	1868.024
		QLSSNFGAISSVLNDILSR	965-983	2022.067	2021.066
		LDPPEAEVQIDR	984-995	1382.709	1381.696
		LQSLQTYVTQQLIR	1001-1014	1692.976	1690.949
		ASANLAATK	1020-1028	847.4766	846.468
		ASANLAATKMSECVLGQSK	1020-1038	1966.989	1965.973
		ASANLAATKMSECVLGQSK	1020-1038	1983.987	1981.968
		MSECVLGQSK	1029-1038	1139.532	1138.523
		MSECVLGQSK	1029-1038	1155.527	1154.518
		RVDFCGK	1039-1045	882.4394	881.4298
		GYHLMSFPQSAPHGVVFLHVTYVPAQEK	1046-1073	3141.618	3139.582
		GYHLMSFPQSAPHGVVFLHVTYVPAQEK	1046-1073	3157.617	3155.577

Identification of proteolytic fragments using mass spectrometry of tryptic peptides.

Glycan masses estimated to be 2.5 kDa. Mass total compared to MWr estimated from migration in gel relative to MW markers.

CS 6P

**S2**	**635-1205**	**Mass (kD)**	**Tryptic peptide**	**P0DTC2**	**MH+ [Da]**	**Theo. MH+ [Da]**
	Chain	71.1	VYSTGSNVFQTR	635-646	1358.671	1358.67
	glycans	17.5	ALTGIAVEQDKNTQEVFAQVK	766-786	2289.205	2289.209
	total	88.6	ALTGIAVEQDK	766-776	1144.62	1144.621
	MWr	114	NTQEVFAQVK	777-786	1163.604	1163.606
			RSPIEDLLFNK	815-825	1331.732	1331.732
			SPIEDLLFNKVTLADAGFIK	816-835	2191.202	2191.201
			SPIEDLLFNK	816-825	1175.63	1175.631
			VTLADAGFIK	826-835	1034.588	1034.588
			QYGDCLGDIAAR	836-847	1281.591	1281.589
			FNGIGVTQNVLYENQK	906-921	1823.927	1823.929
			LIANQFNSAIGK	922-933	1275.707	1275.706
			IQDSLSSTPSALGK	934-947	1403.737	1403.738
			LQDVVNQNAQALNTLVK	948-964	1868.024	1868.024
			QLSSNFGAISSVLNDILSR	965-983	2021.066	2021.066
			LDPPEAEVQIDR	984-995	1381.695	1381.696
			LQSLQTYVTQQLIR	1001-1014	1690.948	1690.949
			MSECVLGQSK	1029-1038	1097.497	1097.497
			GYHLMSFPQSAPHGVVFLHVTYVPAQEK	1046-1073	3155.584	3155.577
			NLNESLIDLQELGK	1192-1205	1585.843	1585.843

**836-1205**	**Mass (kD)**	**Tryptic peptide**	**P0DTC2**	**MH+ [Da]**	**Theo. MH+ [Da]**
Chain	40.5	QYGDCLGDIAAR	836-847	1281.589	1281.589
glycans	5	FNGIGVTQNVLYENQK	906-921	1823.929	1823.929
total	**45.5**	FNGIGVTQNVLYENQKLIANQFNSAIGK	906-933	3080.618	3080.616
MWr (gel)	**58.9**	FNGIGVTQNVLYENQKLIANQFNSAIGKIQDSLSSTPSALGK	906-947	4465.329	4465.336
		LIANQFNSAIGK	922-933	1275.706	1275.706
		LIANQFNSAIGKIQDSLSSTPSALGK	922-947	2660.425	2660.425
		IQDSLSSTPSALGK	934-947	1403.736	1403.738
		IQDSLSSTPSALGKLQDVVNQNAQALNTLVK	934-964	3252.738	3252.743
		LQDVVNQNAQALNTLVK	948-964	1868.023	1868.024
		QLSSNFGAISSVLNDILSR	965-983	2021.065	2021.066
		AAEIRASANLAATK	1015-1028	1386.771	1386.77
		ASANLAATK	1020-1028	846.4676	846.468
		MSECVLGQSK	1029-1038	1097.496	1097.497
		GYHLMSFPQSAPHGVVFLHVTYVPAQEK	1046-1073	3155.575	3155.577
		EELDKYFK	1150-1157	1071.535	1071.536
		EIDRLNEVAK	1182-1191	1186.644	1186.643
		LNEVAK	1186-1191	673.3883	673.3879
		NLNESLIDLQELGK	1192-1205	1585.842	1585.843

Identification of proteolytic fragments using mass spectrometry of tryptic peptides.

Glycan masses estimated to be 2.5 kDa. Mass total compared to MWr estimated from migration in gel relative to MW markers.

PK 2P

**S2**	**719-1260**	**Mass totals**	**Peptide**	**P0DTC2**	**MH+ [Da]**	**Theo. MH+ [Da]**
	Chain mas	59.7	[F].TISVTTEILPVSMTK	719-733	1637.903	1635.887
	glycans	14	TSVDCTMYICGDSTECSNLLLQYGSFCTQLNR	735-766	3795.609	3793.627
	total	73.7	ALTGIAVEQDK	767-777	1145.628	1144.621
	MWr (gel)	91.2	ALTGIAVEQDKNTQEVFAQVK	767-787	2290.199	2289.209
			ALTGIAVEQDKNTQEVFAQVKQIYK	767-791	2823.535	2821.509
			NTQEVFAQVK	778-787	1164.613	1163.606
			NTQEVFAQVKQIYK	778-791	1696.919	1695.906
			DFGGFNFSQILPDPSKPSK	797-815	2083.055	2081.034
			DFGGFNFSQILPDPSKPSKR	797-816	2240.159	2237.135
			RSFIEDLLFNK	816-826	1382.758	1381.747
			SFIEDLLFNK	817-826	1226.654	1225.646
			SFIEDLLFNKVTLADAGFIK	817-836	2242.222	2241.217
			SFIEDLLFNKVTLADAGFIKQYGDCLGDIAAR	817-848	3563.829	3560.809
			VTLADAGFIK	827-836	1035.595	1034.588
			VTLADAGFIKQYGDCLGDIAAR	827-848	2356.198	2354.181
			QYGDCLGDIAAR	837-848	1339.62	1338.611
			DLICAQK	849-855	848.4423	847.4342
			FNGIGVTQNVLYENQK	907-922	1825.946	1823.929
			FNGIGVTQNVLYENQKLIANQFNSAIGK	907-934	3082.632	3080.616
			FNGIGVTQNVLYENQKLIANQFNSAIGKIQDSLSSTASALGK	907-948	4442.345	4439.321
			LIANQFNSAIGK	923-934	1276.715	1275.706
			LIANQFNSAIGKIQDSLSSTASALGK	923-948	2636.433	2634.41
			LIANQFNSAIGKIQDSLSSTASALGKLQDVVNQNAQALNTLVK	923-965	4486.42	4483.415
			IQDSLSSTASALGK	935-948	1378.73	1377.722
			IQDSLSSTASALGKLQDVVNQNAQALNTLVK	935-965	3228.743	3226.728
			LQDVVNQNAQALNTLVK	949-965	1869.034	1868.024
			QLSSNFGAISSVLNDILSR	966-984	2022.072	2021.066
			QLSSNFGAISSVLNDILSRLDPPEAEVQIDR	966-996	3385.74	3383.744
			LDPPEAEVQIDR	985-996	1383.712	1381.696
			LITGRLQSLQTYVTQQLIR	997-1015	2233.306	2231.287
			LITGRLQSLQTYVTQQLIRAAEIR	997-1020	2774.617	2771.589
			LQSLQTYVTQQLIR	1002-1015	1692.967	1690.949
			LQSLQTYVTQQLIRAAEIR	1002-1020	2233.273	2231.251
			AAEIRASANLAATK	1016-1029	1388.785	1386.77
			ASANLAATK	1021-1029	847.477	846.468
			ASANLAATKMSECVLGQSK	1021-1039	1966.983	1965.973
			ASANLAATKMSECVLGQSK	1021-1039	1983.982	1981.968
			MSECVLGQSK	1030-1039	1139.532	1138.523
			MSECVLGQSK	1030-1039	1155.527	1154.518
			RVDFCGK	1040-1046	882.439	881.4298
			GYHLMSFPQSAPHGVVFLHVTYVPAQEK	1047-1074	3141.602	3139.582
			GYHLMSFPQSAPHGVVFLHVTYVPAQEK	1047-1074	3157.602	3155.577
			NFTTAPAICHDGK	1075-1087	1433.683	1431.669
			EGVFVSNGTHWFVTQR	1093-1108	1865.933	1863.914
			NFYEPQIITTDNTFVSGNCDVVIGIVNNTVYDPLQPELDSFK	1109-1150	4779.307	4776.303
			NHTSPDVDLGDISGINASVVNIQK	1159-1182	2495.269	2493.258
			EIDRLNEVAK	1183-1192	1187.652	1186.643
			LNEVAKNLNESLIDLQELGK	1187-1206	2242.232	2240.213
			NLNESLIDLQELGK	1193-1206	1586.853	1585.843
			NLNESLIDLQELGKYEQGSGYIPEAPR	1193-1219	3035.537	3033.516
			YEQGSGYIPEAPR	1207-1219	1467.7	1466.691
			YEQGSGYIPEAPRDGQAYVRK	1207-1227	2385.169	2384.163
			DGQAYVR	1220-1226	809.4044	808.3948
			DGQAYVRK	1220-1227	937.4976	936.4898
			DGQAYVRKDGEWVLLSTFLGR	1220-1240	2412.271	2410.251
			KDGEWVLLSTFLGR	1227-1240	1621.884	1620.874
			DGEWVLLSTFLGR	1228-1240	1493.776	1492.779
			SLEVLFQGPGHHHHHHHHSAW.[S]	1241-1260	2490.194	2487.171

**719-1135**	**Mass total**	**Peptide**	**P0DTC2**	**MH+ [Da]**	**Theo. MH+ [Da]**
Chain mas	45.5	[F].TISVTTEILPVSMTK	719-733	1636.897	1635.887
glycans	6	ALTGIAVEQDKNTQEVFAQVK	766-786	2291.224	2289.209
total	57.5	ALTGIAVEQDK	766-776	1145.628	1144.621
MWr	72.9	NTQEVFAQVK	777-786	1164.61	1163.606
		NTQEVFAQVKQIYK	777-790	1696.911	1695.906
		DFGGFNFSQILPDPSKPSK	796-814	2083.048	2081.034
		RSFIEDLLFNK	815-825	1382.759	1381.747
		SFIEDLLFNK	816-825	1226.653	1225.646
		SFIEDLLFNKVTLADAGFIK	816-835	2243.233	2241.217
		VTLADAGFIK	826-835	1035.595	1034.588
		VTLADAGFIKQYGDCLGDIAAR	826-847	2356.206	2354.181
		QYGDCLGDIAAR	836-847	1339.618	1338.611
		FNGIGVTQNVLYENQK	906-921	1824.936	1823.929
		FNGIGVTQNVLYENQKLIANQFNSAIGK	906-933	3082.635	3080.616
		LIANQFNSAIGK	922-933	1276.712	1275.706
		LIANQFNSAIGKIQDSLSSTASALGK	922-947	2636.429	2634.41
		IQDSLSSTASALGK	934-947	1378.73	1377.722
		IQDSLSSTASALGKLQDVVNQNAQALNTLVK	934-964	3228.742	3226.728
		LQDVVNQNAQALNTLVK	948-964	1870.045	1868.024
		QLSSNFGAISSVLNDILSR	965-983	2023.081	2021.066
		LDPPEAEVQIDR	984-995	1382.702	1381.696
		LQSLQTYVTQQLIR	1001-1014	1691.961	1690.949
		ASANLAATK	1020-1028	847.4756	846.468
		ASANLAATKMSECVLGQSK	1020-1038	1983.983	1981.968
		MSECVLGQSK	1029-1038	1155.526	1154.518
		RVDFCGK	1039-1045	882.4379	881.4298
		GYHLMSFPQSAPHGVVFLHVTYVPAQEK	1046-1073	3158.623	3155.577
		NFTTAPAICHDGK	1074-1086	1432.677	1431.669
		[N].TVYDPLQPELDSFKEELDK	1136-1154	2268.131	2266.113

Identification of proteolytic fragments using mass spectrometry of tryptic peptides.

Glycan masses estimated to be 2.5 kDa. Mass total compared to MWr estimated from migration in gel relative to MW markers.

PK 6P

**S2**	**635-1205**	**Mass (kD)**	**Tryptic peptide**	**P0DTC2**	**MH+ [Da]**	**Theo. MH+ [Da]**
	Chain	62.4	VYSTGSNVFQTR	635-646	1358.669	1358.67
	glycans	17.5	ALTGIAVEQDK	766-776	1144.62	1144.621
	total	79.9	ALTGIAVEQDK	766-776	1144.62	1144.621
	MWr (gel)	78.7	NTQEVFAQVK	777-786	1163.604	1163.606
			RSPIEDLLFNK	815-825	1331.733	1331.732
			SPIEDLLFNK	816-825	1175.631	1175.631
			SPIEDLLFNKVTLADAGFIK	816-835	2191.197	2191.201
			VTLADAGFIK	826-835	1034.588	1034.588
			FNGIGVTQNVLYENQK	906-921	1823.93	1823.929
			LIANQFNSAIGK	922-933	1275.708	1275.706
			IQDSLSSTPSALGK	934-947	1403.738	1403.738
			LQDVVNQNAQALNTLVK	948-964	1868.019	1868.024
			QLSSNFGAISSVLNDILSR	965-983	2021.066	2021.066
			LDPPEAEVQIDR	984-995	1381.695	1381.696
			LQSLQTYVTQQLIR	1001-1014	1690.946	1690.949
			GYHLMSFPQSAPHGVVFLHVTYVPAQEK	1046-1073	3155.57	3155.577
			NLNESLIDLQELGK	1192-1205	1585.843	1585.843

**766-1014**	**Mass (kD)**	**Tryptic peptide**	**P0DTC2**	**MH+ [Da]**	**Theo. MH+**
Chain	27.1	ALTGIAVEQDKNTQEVFAQVK	766-786	2290.221	2288.201
glycans	2.5	ALTGIAVEQDK	766-776	1145.63	1143.614
total	29.6	VTLADAGFIK	826-835	1035.599	1033.581
MWr	34.5	FNGIGVTQNVLYENQK	906-921	1824.942	1822.921
		LIANQFNSAIGK	922-933	1276.717	1274.698
		IQDSLSSTASALGK	934-947	1378.735	1376.715
		LQDVVNQNAQALNTLVK	948-964	1869.039	1867.016
		LQSLQTYVTQQLIR	1001-1014	1692.971	1689.941

**766-1086**	**Mass (kD)**	**Tryptic peptide**	**P0DTC2**	**MH+ [Da]**	**Theo. MH+**
Chain	34.8	ALTGIAVEQDKNTQEVFAQVK	766-786	2289.195	2289.209
glycans	5	NTQEVFAQVK	777-786	1163.603	1163.606
total	39.8	NTQEVFAQVKQIYK	777-790	1695.906	1695.906
MWr	67.7	VTLADAGFIK	826-835	1034.589	1034.588
		QYGDCLGDIAAR	836-847	1338.611	1338.611
		DLICAQK	848-854	847.4339	847.4342
		FNGIGVTQNVLYENQK	906-921	1823.931	1823.929
		LIANQFNSAIGK	922-933	1275.707	1275.706
		LQDVVNQNAQALNTLVK	948-964	1868.025	1868.024
		QLSSNFGAISSVLNDILSR	965-983	2021.066	2021.066
		LITGRLQSLQTYVTQQLIR	996-1014	2231.286	2231.287
		LQSLQTYVTQQLIR	1001-1014	1690.946	1690.949
		AAEIRASANLAATK	1015-1028	1386.769	1386.77
		ASANLAATK	1020-1028	846.4673	846.468
		MSECVLGQSK	1029-1038	1154.518	1154.518
		MSECVLGQSKR	1029-1039	1310.618	1310.619
		RVDFCGK	1039-1045	881.4297	881.4298
		GYHLMSFPQSAPHGVVFLHVTYVPAQEK	1046-1073	3155.567	3155.577
		NFTTAPAICHDGK	1074-1086	1431.669	1431.669

Table S3. SARS-CoV-2 peptide pools 9S and 9U.

	Peptide	APL	Residue range	Omicron BA.1 cross-reaction
**Peptide 19 (9S)**	**VIKVCEFQFCNDPFLGV**	0.49	127-143	
Omicron BA.1	···············D-			
Omicron BA.2	···············D·			
Omicron JN.1	F··············D·			
CCCV OC43	EVS··QYNM·EY·QTIC			
**Peptide 24 (9U)**	**SANNCTFEYVSQPFLMD**	0.06	162-178	
Omicron BA.1	·················			
Omicron BA.2	·················			
Omicron JN.1	·················			
CCCV OC43	-LYKRN·T·DVN--A--			
**Peptide 28 (9S)**	**REFVFKNIDGYFKIYSK**	0.27	190-206	
Omicron BA.1	·················			
Omicron BA.2	·················			
Omicron JN.1	·················			
CCCV OC43	-Y·H·YQEG·T·YA·FT			
**Peptide 30 (9U)**	**YSKHTPINLVRDLPQGF**	0.17	204-220	
Omicron BA.1	·······-I········			Poor (9)
	+EPE			
Omicron BA.2	·········G·······			
Omicron JN.1	·······-IG··F····			
CCCV OC43	·FTD·----------·V			
**Peptide 31 (9U)**	**NLVRDLPQGFSALEPLV**	0.12	211-227	
Omicron BA.1	-I···············			Poor (9)
	+EPE			
Omicron BA.2	··G··············			
Omicron JN.1	-IG··F···········			
CCCV OC43	--------·VV-TKF·F			
**Peptide 50 (9S)**	**ATRFASVYAWNRKRISN**	0.27	344-360	
Omicron BA.1	·················			
Omicron BA.2	·················			
Omicron JN.1	············T····			
CCCV OC43	DKSVP·PLN·E··TF··			
**Peptide 51 (9S)**	**YAWNRKRISNCVADYSV**	0.45	351-367	
Omicron BA.1	·················			
Omicron BA.2	·················			
Omicron JN.1	·····T···········			
CCCV OC43	LN·E··TF···NFNM·S			
**Peptide 55 (9S)**	**CYGVSPTKLNDLCFTNV**	0.44	379-395	
Omicron BA.1	·················			
Omicron BA.2	·················			
Omicron JN.1	·················			
CCCV OC43	·NNIDAA·IYGM··SSI			
**Peptide 64 (9U)**	**DSKVGGNYNYLYRLFRK**	0.06	442-458	
Omicron BA.1	····S············			Unaffected (9)
Omicron BA.2	·················			
Omicron JN.1	···HS···D·W··S···			
CCCV OC43	NVS·SR-F·PSTWNK·F			
**Peptide 65 (9U)**	**YNYLYRLFRKSNLKPFE**	0.05	449-465	
Omicron BA.1	·················			
Omicron BA.2	·················			
Omicron JN.1	·D·W··S····K·····			
CCCV OC43	F·PSTWNK·FGFIEDHD			
**Peptide 74 (9U)**	**VLSFELLHAPATVCGPK**	0.07	512-528	
Omicron BA.1	·················			
Omicron BA.2	·················			
Omicron JN.1	·················			
CCCV OC43	F·GWANFVNSGLT·ST·			

Table S3. SARS-CoV-2 peptide pools 9S and 9U (cont'd).

	Peptide	APL	Residue range	Omicron BA.1 cross-reaction
**Peptide 104 (9S)**	**VTTEILPVSMTKTSVDC**	0.70	722-738	
Omicron BA.1	·················			
Omicron BA.2	·················			
Omicron JN.1	·················			
CCCV OC43	NMV·FIQT·SP·VTI··			
**Peptide 107 (9U)**	**CGDSTECSNLLLQYGSF**	0.19	743-759	
Omicron BA.1	·················			
Omicron BA.2	·················			
Omicron JN.1	·················			
CCCV OC43	···YAA·KSQ·VE····			
**Peptide 108 (9U)**	**SNLLLQYGSFCTQLNRA**	0.25	750-766	
Omicron BA.1	··············K··			Poor (9)
Omicron BA.2	··············K··			
Omicron JN.1	··············K··			
CCCV OC43	KSQ·VE·····DNI·AI			
**Peptide 115 (9U)**	**GFNFSQILPDPSKPSKR**	0.17	799-815	
Omicron BA.1	·················			
Omicron BA.2	·················			
Omicron JN.1	·················			
CCCV OC43	DD···PV·GLG··A·S·			
**Peptide 124 (9S)**	**PPLLTDEMIAQYTSALL**	0.50	862-878	
Omicron BA.1	·················			
Omicron BA.2	·················			
Omicron JN.1	·················			
CCCV OC43	····SENQ·SG··L·AT			
**Peptide 125 (9S)**	**MIAQYTSALLAGTITSG**	0.44	869-885	
Omicron BA.1	·················			
Omicron BA.2	·················			
Omicron JN.1	·················			
CCCV OC43	Q·SG··L·ATSASLFPP			
**Peptide 145 (9S)**	**TQQLIRAAEIRASANLA**	0.53	1009-1025	
Omicron BA.1	·················			
Omicron BA.2	·················			
Omicron JN.1	·················			
CCCV OC43	S···SDSTLVKF··AQ·			

Table S4. Peptide pairs used for partial deconvolution of responses to 9S and 9U peptide pools. Sequences within the reported epitopes were predicted to have sufficient affinity for the indicated alleles to present the epitopes. The Calculated Panel Reactive Antibodies (CPRA) score combines the frequency of the listed alleles in the US population to calculate the proportion of individuals having any one of the alleles. [https://optn.transplant.hrsa.gov/data/allocation-calculators/cpra-calculator/]

**50-51**	Reported epitopes	Restricting allele	Reference	Frequency	CPRA
ATRFASVYAWNRKRISN					99%
(344-360)					
YAWNRKRISNCVADYSV					
(351-367)					
	WNRKRISNCVADYSV	DRB1*03:01	(3)	0.1925425	
	(353–367)				
	VYAWNRKRISNCVAD	Multiple DR and DP	(4)		
	(346-365)				
	TRFASVYAWNRKRISNCVAD	DRB1*03:01	(5)	0.1135415	
	(345-364)	DRB1*11:01		0.280939	
		DRB5			
	VFNATRFASVYAWNR	DRB1*01:01	(6)	0.1263433	
	(341-355)	DRB1*08:03		0.0061661	
		DRB1*11:01		0.1135415	
		DRB1*13:03		0.0295027	
		DRB1*15:01		0.1915606	
		DQA1*05:01	-	0.4124896	
		DQB1*03:01		0.3164956	
		DQA1*01:02	-	0.3579131	
		DQB1*06:02		0.2348541	
	RFASVYAWNRKRISN	DRB1*07:01	(7)	N/A	
	(346-360)	DRB1*13:01		0.1097306	
		DRB1*14:01		0.0481988	
	YAWNRKRISNCVADY	DRB1*13:01		0.1097306	
	(351-365)				

**64-65**	Reported epitopes	Restricting allele	Reference	Frequency	CPRA
DSKVGGNYNYLYRLFRK					52%
(442-458)					
YNYLYRLFRKSNLKPFE					
(449-465)					
	VGGNYNYLYRLFRKS	DRB1*11:01	(3)	0.1135415	
	(445–459),	DRB1*16:01		0.0232932	
	YNYLYRLFRKSNLKP	DRB1*04:05		0.0217074	
	(449–463)	DRB1*13:01		0.1097306	
		DRB1*15:01		0.1915606	
		DRB1*16:01		0.0232932	
	LDSKVGGNYNYLYRLFRKSN	DRB1*11:01	(5)	0.1135415	
	(441-460)	DRB1*11:04		0.0574157	
	GGNYNYLYRLFRKSN	DRB1*11:02	(6)	N/A	
	(446-460)	DRB1*13:03		0.0295027	

**107-108**	Reported epitopes	Restricting allele	Reference	Frequency	CPRA
CGDSTECSNLLLQYGSF					52%
(743-759)					
SNLLLQYGSFCTQLNRA					
(750-766)					
	DSTECSNLLLQYGSFCTQLN	DRB1*15:01	(5)	0.1915606	
	(745-764)				
	LLQYGSF	DRB1*15:01	(8)	0.1915606	
	(753-759)				
	NLLLQYGSFCTQLNR	DRB1*01:01	(6)	0.1263433	
	(751-765)	DRB1*15:01		0.1915606	
		DQA1*01:01		0.2601797	
		DQB1*05:01			
	STECSNLLLQYGSFC	DQB1*06:02	(7)	0.2348541	
	(746-760)				
	NLLLQYGSFCTQLNR	DQB1*05:03		0.0507113	
	(751-765)	DRB1*04:04		0.0646639	
		DRB1*15:01		0.1915606	

**124-125**	Reported epitopes	Restricting allele	Reference	Frequency	CPRA
PPLLTDEMIAQYTSALL					69%
(862-878)					
MIAQYTSALLAGTITSG					
(869-885)					
	GLTVLPPLLTDEMIAQYTSA	DRB1*03:01	(5)	0.1925425	
	(857-876)				
	LTDEMIAQYTSALLAGTITS	DRB1*15:01		0.1915606	
	(865-884)				
	MIAQYTSALLA	DRB1*15:01	(8)	0.1915606	
	(869-879)	DRB1*15:02		0.0213748	
		DRB1*15:03		0.039292	
		DRB1*15:04		N/A	
		DRB1*15:06		N/A	
		DRB1*16:01		0.0232932	
		DRB1*16:02		0.0140534	
		DQA1*01:01		0.2601797	
		DQB1*05:01		0.2245781	
		DQA1*01:05		0.2601797	
		DQB1*05:01		0.2245781	
	TDEMIAQYTSALLAG	DRB1*0410	(6)	0.0011071	
	(866-880)	DRB1*0701		N/A	
		DQA1*0201-DQB1*0202		0.250033	
		DQA1*0301- DQB1*0302		0.204127	
	LPPLLTDEMIAQYTS	DQB1*04:02	(7)	0.0861561	
	(861-875)				
	TDEMIAQYTSALLAG	DQB1*06:02		0.2348541	
	(866-880)	DRB1*04:04		0.0646639	
		DRB1*15:01		0.1915606	
	AQYTSALLAGTITSG	DQB1*06:02		0.2348541	
	(871-885)				

Table S5 (Related to Fig. 3): Cohort characteristics

	Infected + Vac		Infected only	
Date range	2020-11-25	2021-05-25	2020-06-17	2021-05-25
Subjects		18		34
Male		8		15
Female		10		19
Average Age		52.3		40.9
Avg infections		1		34
Average Vac injections		1.7(1 or 2)		0

Inclusion criteria:
History of infection
Record of vaccination (where indicated)
Minimum 1.5e6 PBMCs
>0 raw 9U spots
Exclusion criteria:
Immunocompromised
HIV and CD4+ cells < 500 per µL

Table S6 (Related to Fig. 4): Cohort characteristics

	Infected +/- Vac		Vac only	
Date range	2020-06-12	2021-11-03	2021-02-02	2021-12-14
Subjects		54		17
Male		18		5
Female		36		12
Average Age		41.3		46.8
Avg Infections		1		0
Avg Vac injection		1.2(0,1,2, or 3)		2.1(1, 2, or 3)

Inclusion criteria:
History of infection or record of vaccination
minimum 1.5e6 cells
>0 raw 9U spots
Exclusion criteria:
Immunocompromised
HIV and CD4+ cells < 500 per µL

Table S7 (Related to Fig. 5): Cohort characteristics

	Infected + Vac		
Date range	5/26/2023	8/11/2023	
Subjects		20	
Male		11	
Female		9	
Average Age		59.0	
Avg Infections			1.5(1, 2, or 3)
Avg Vac injection			3.3(1-7)
<u>Inclusion criteria:</u>			
History of infection			
minimum 1.5e6 cells			
>0 raw 9U spots			
<u>Exclusion criteria:</u>			
Immunocompromised			
HIV and CD4+ cells < 500 per μL			

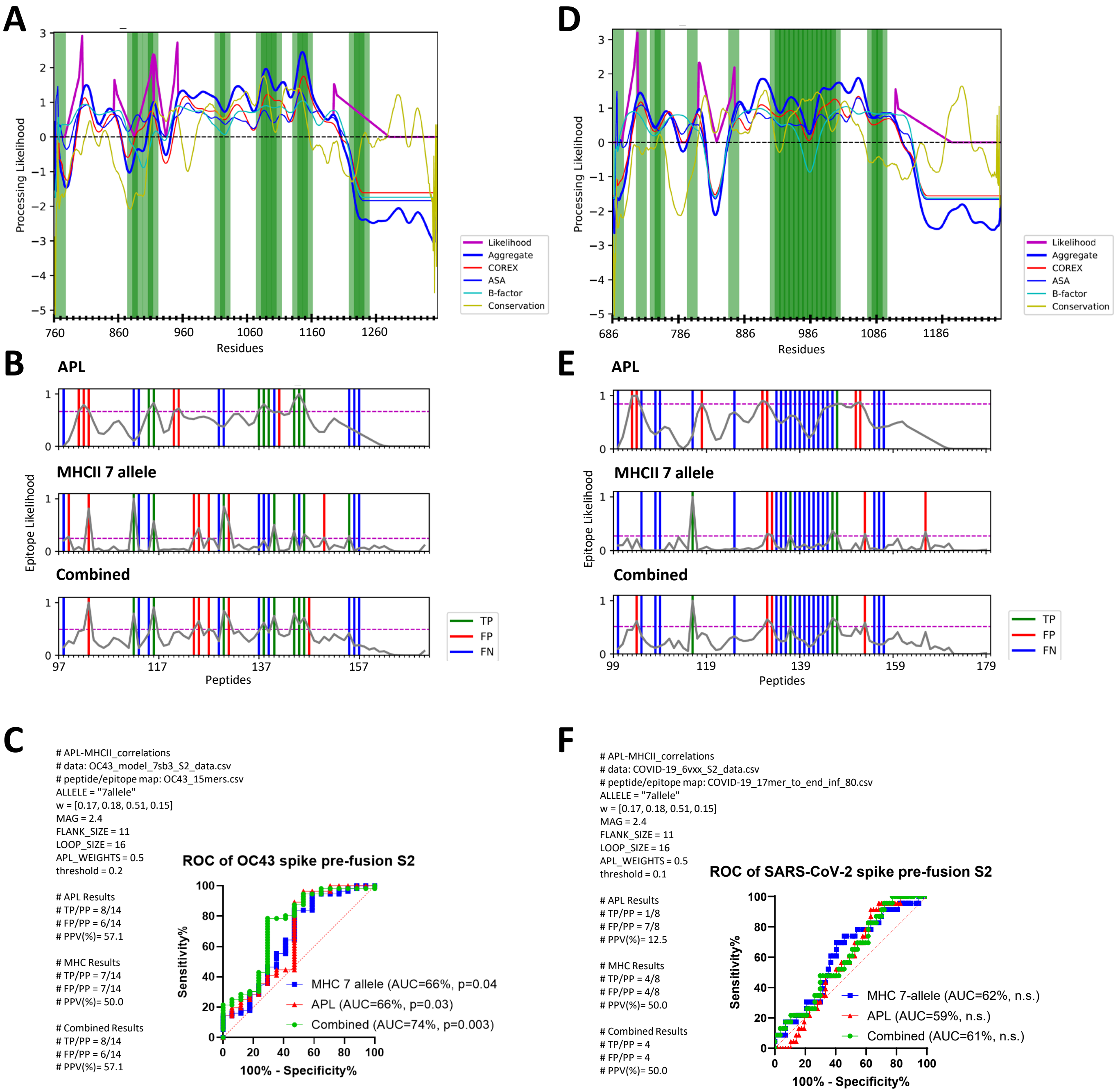

Figure S1. Performance of Epitope Predictions for OC43 (A-C) and SARS-CoV-2 (D-F) Spike pre-fusion S2. In A and D, APL by residue with reported epitopes (vertical bars). In B and E, APL, MHCII binding, and Combined predictions by peptide with true-positive (TP), false-positive (FP), and false-negative (FN) peptides indicated. In C and F, summary of APL parameters and accuracy. Modest accuracy was obtained for OC43 S2 and was improved by combining APL and MHCII binding.

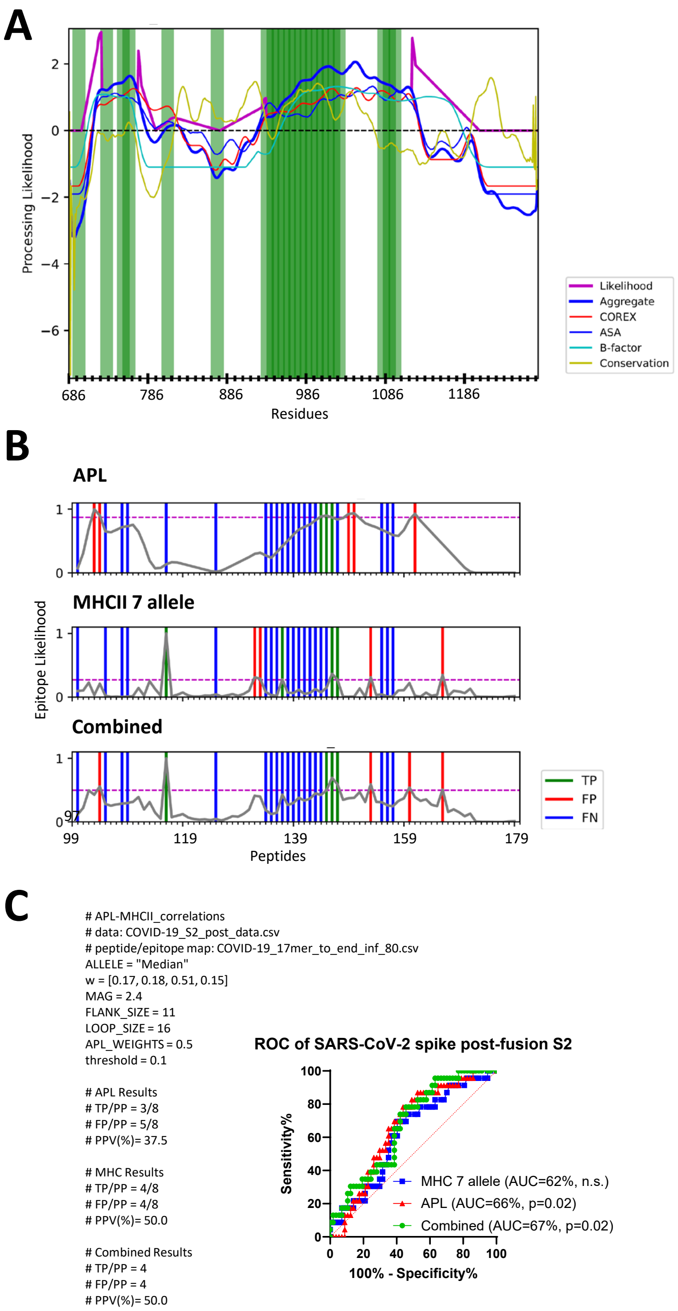

Figure S2. Performance of Epitope Predictions for SARS-CoV-2 Spike post-fusion S2. In A, APL by residue with reported epitopes (vertical bars). In B, APL, MHCII binding, and Combined predictions by peptide with true-positive (TP), false-positive (FP), and false-negative (FN) peptides indicated. In C, summary of APL parameters and accuracy. Modest accuracy was obtained by APL.

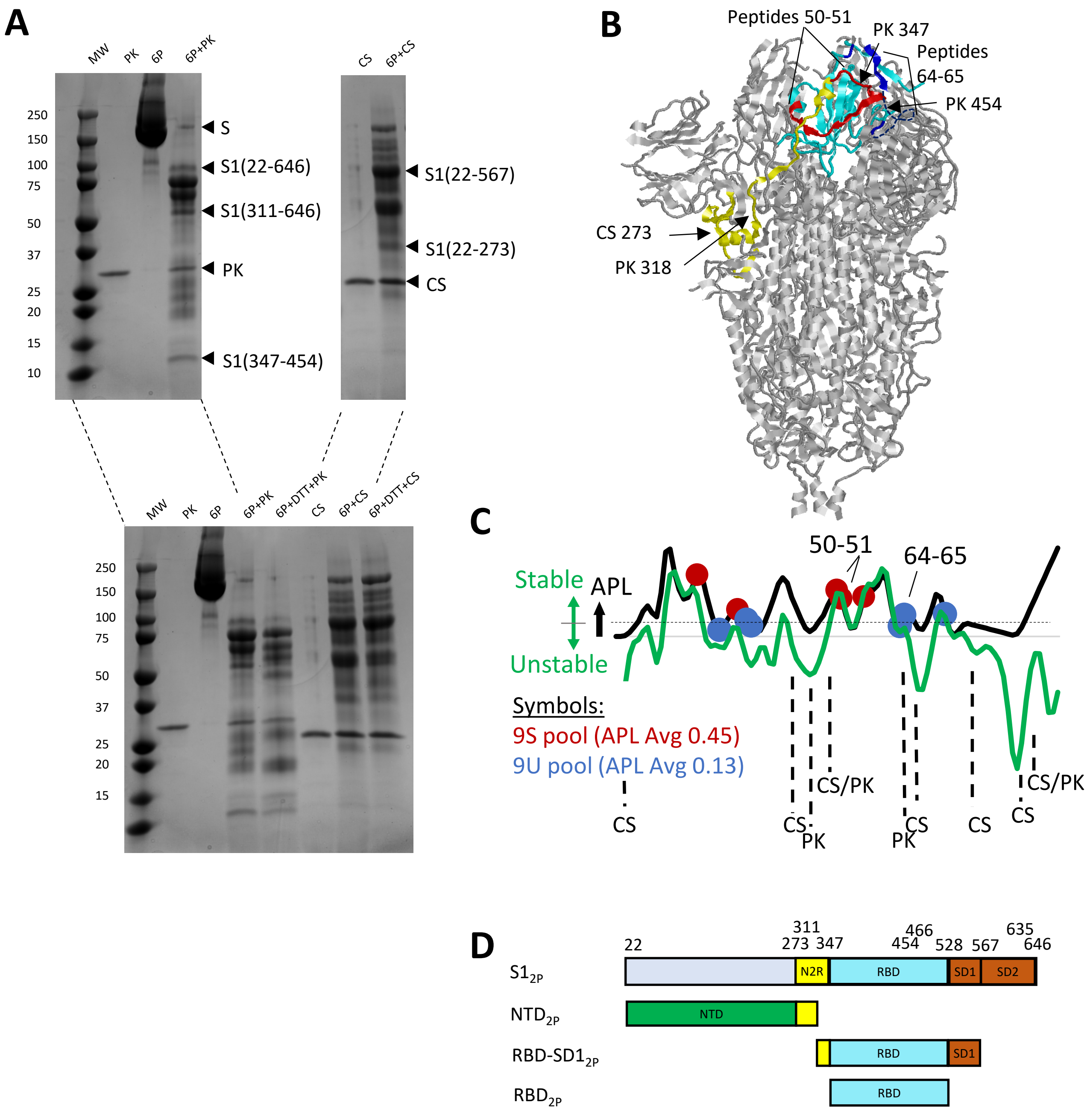

Figure S3. Spike-6P S1 conformationally unstable, solvent-exposed, protease-sensitive regions. A. Coomassie-stained bands generated by limited proteolysis of recombinant spike-6P with cathepsin S (CS) or proteinase K (PK), separated by SDS-PAGE, and identified by tryptic digestion and mass spectrometry. Banding patterns are typical of at least three experiments. B. Ribbon diagram of the intact spike prefusion trimer (PDB: 6VXX), indicating the dominant epitope-containing peptides and flanking protease cleavage sites in S1. C. APL and aggregate stability for S2 annotated as in Fig. 1A. D. Diagram of major proteolytic fragments in S1 generated by limited proteolysis of spike-6P with CS (1.8, 0.9, or 0.45 µg) or PK (1.5, 0.06, or 0.03 µg).

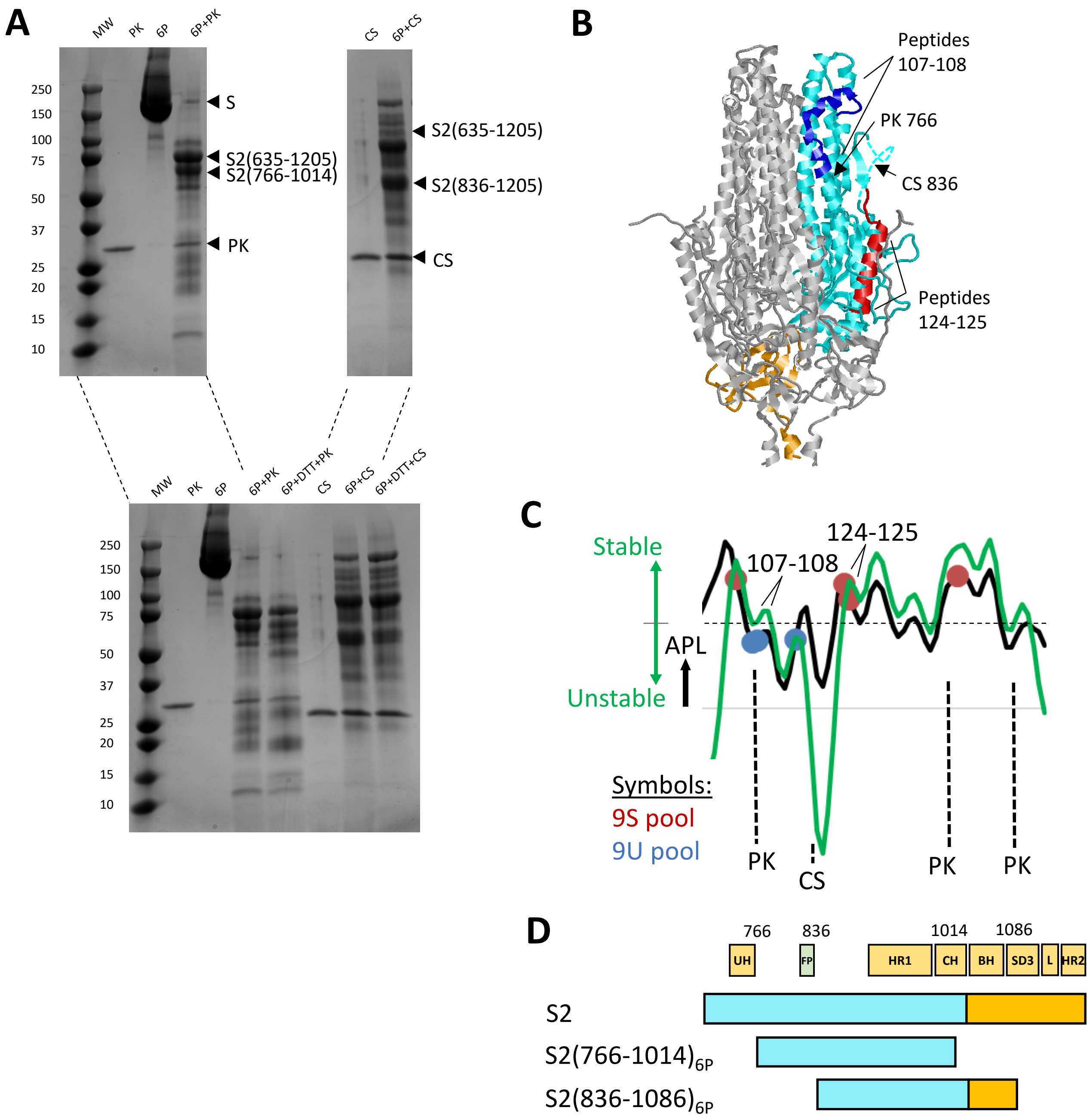

Figure S4. Spike-6P S2 conformationally unstable, solvent-exposed, protease-sensitive regions. A. Coomassie-stained bands generated by limited proteolysis of recombinant spike-6P with cathepsin S (CS) or proteinase K (PK), separated by SDS-PAGE, and identified by tryptic digestion and mass spectrometry. Banding patterns are typical of at least three experiments. B. Ribbon diagram of the intact spike prefusion trimer (PDB: 6VXX), indicating the dominant epitope-containing peptides and flanking protease cleavage sites in S1. C. APL and aggregate stability for S2 annotated as in Fig. 1A. Structural domains are as follows (Ref. 10): upstream helix (UH), fusion peptide (FP), heptad repeat 1 (HR1), central helix (CH), beta-hairpin (BH), subdomain 3 (SD3), linker (L), and heptad repeat 2 (HR2). D. Diagram of major proteolytic fragments in S2 generated by limited proteolysis of spike-6P with CS (1.8, 0.9, or 0.45 µg) or PK (1.5, 0.06, or 0.03 µg). E. Ribbon diagram of the S2 portion of the intact prefusion trimer (PDB: 6VXX), indicating the dominant epitope-containing peptides and flanking protease cleavage sites in S2.

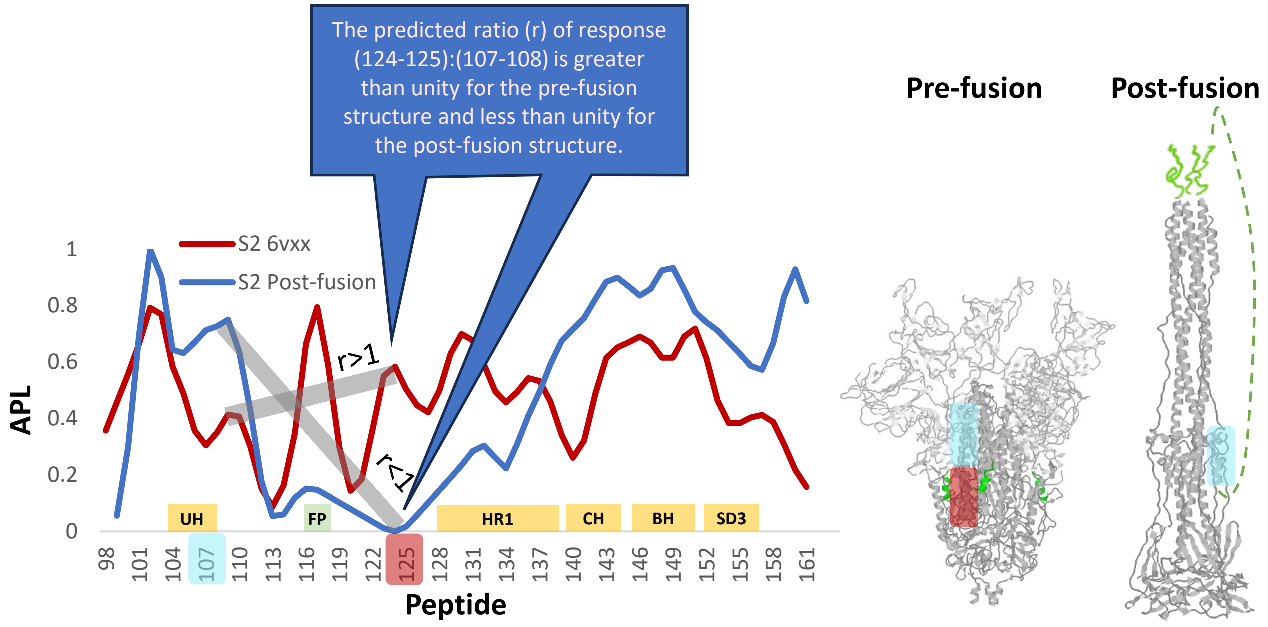

Figure S5. Comparison of APL profiles for the SARS-CoV-2 spike S2 in the pre-fusion conformation (PDB: 6VXX) and post-fusion conformation (modeled on PDB: 6B3O). The S2 segment spanning peptides 109-128 (residues 770-912) is illustrated as a broken line. The fusion peptide (green) is resolved in the pre-fusion structure and indicated by its approximate location in the post-fusion structure. Segments corresponding to peptides 107-108 (cyan highlight) are resolved in both structures, but the segment corresponding to peptides 124-125 (red highlight) is resolved only in the pre-fusion structure. Structural domains are as follows (Ref. 10): upstream helix (UH), fusion peptide (FP), heptad repeat 1 (HR1), central helix (CH), beta-hairpin (BH), and subdomain 3 (SD3).

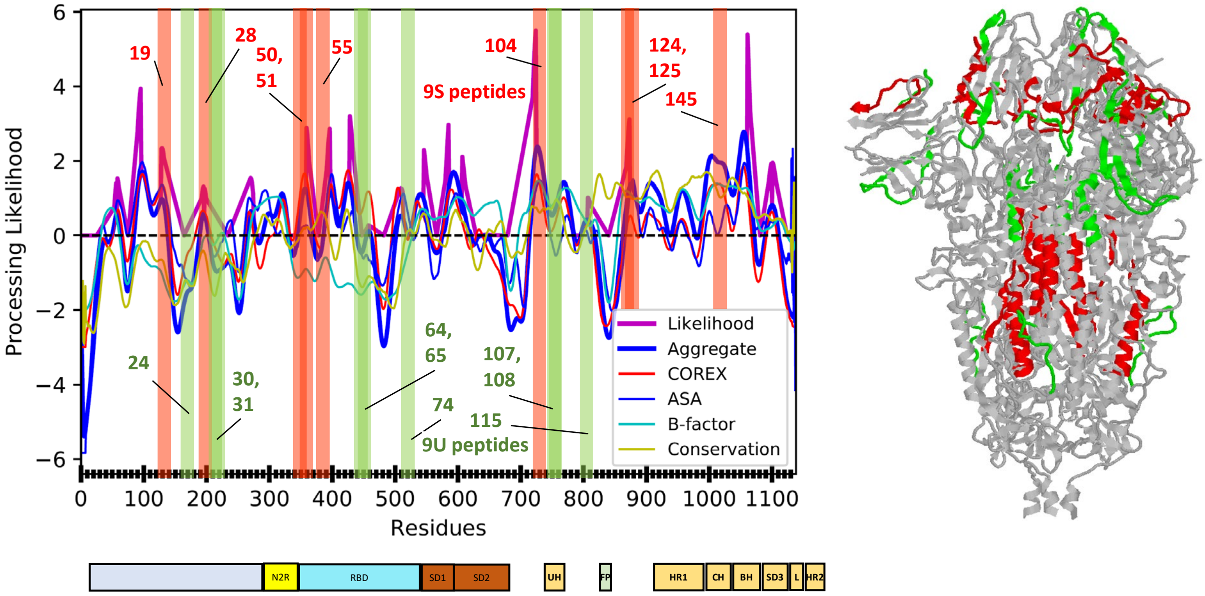

Figure S6. Evaluation of Antigen Processing Likelihood (APL) for the SARS-CoV-2 spike (Wuhan strain) based on the "all-down" conformation (PDB: 6VXX) of receptor-binding domains and selection of 9S and 9U peptide pools. Conservation, B-factor, solvent-accessible surface area (ASA), COREX, and Aggregate stability are presented as the Z-score deviation from average for the entire chain. APL ranges from zero at positions of negative Aggregate stability to 2.4-times the value of aggregate stability near unstable segments that have negative Aggregate stability of 16 residues or more (putative proteolytic processing sites).

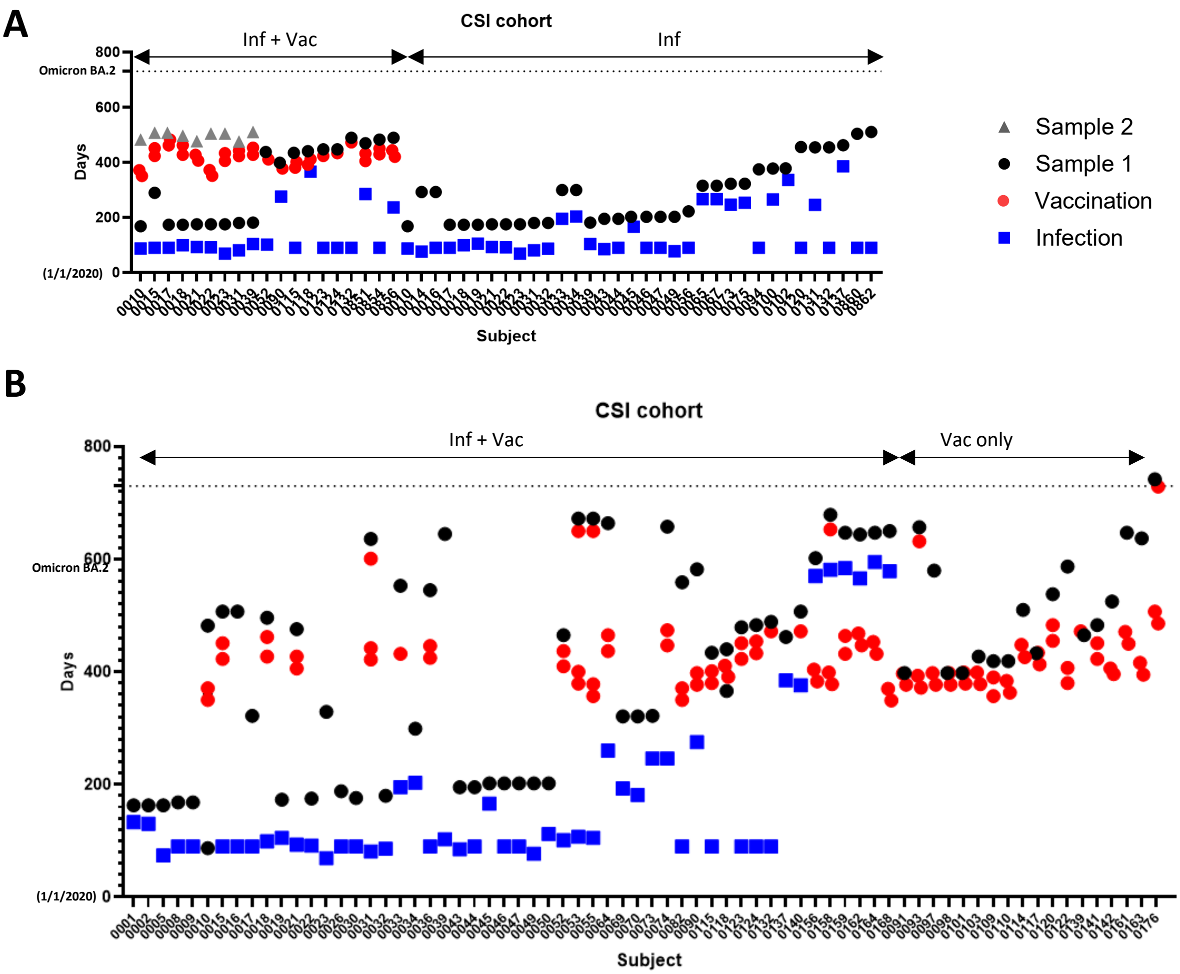

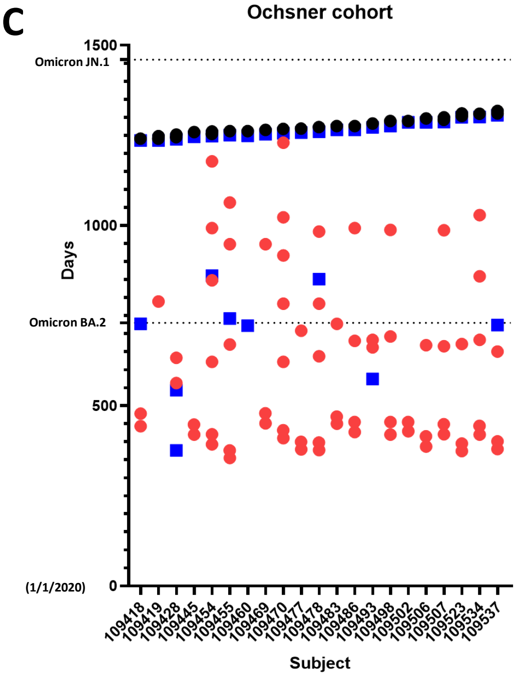

Figure S7. Human-subject timelines. A. Subjects related to Fig. 3. B. Subjects related to Fig. 4. C. Subjects related to Fig. 5. Symbols represent dates of infections (blue squares), mRNA vaccination shots (red circles), blood sample #1 (black circle), or blood sample #2, (gray triangle when paired or black circle). All timelines are counted in days from January 1, 2020. Approximate dates for emergence of SARS-CoV-2 strains BA.2 and JN.1 are indicated.

References

1. Zhao, M. M., Zhu, Y., Zhang, L., Zhong, G., Tai, L., Liu, S. et al. (2022) Novel cleavage sites identified in SARS-CoV-2 spike protein reveal mechanism for cathepsin L-facilitated viral infection and treatment strategies *Cell Discov* **8**, 53

2. Verhagen, J., van der Meijden, E. D., Lang, V., Kremer, A. E., Volkl, S., Mackensen, A. *et al.* (2021) Human CD4(+) T cells specific for dominant epitopes of SARS-CoV-2 Spike and Nucleocapsid proteins with therapeutic potential *Clin Exp Immunol* **205**, 363-378

4. Low, J. S., Vaqueirinho, D., Mele, F., Foglierini, M., Jerak, J., Perotti, M. *et al.* (2021) Clonal analysis of immunodominance and cross-reactivity of the CD4 T cell response to SARS-CoV-2 *Science* **372**, 1336-1341

5. Johansson, A. M., Malhotra, U., Kim, Y. G., Gomez, R., Krist, M. P., Wald, A. *et al.* (2021) Cross-reactive and mono-reactive SARS-CoV-2 CD4+ T cells in prepandemic and COVID-19 convalescent individuals *PLoS Pathog* **17**, e1010203

6. Mateus, J., Grifoni, A., Tarke, A., Sidney, J., Ramirez, S. I., Dan, J. M. *et al.* (2020) Selective and cross-reactive SARS-CoV-2 T cell epitopes in unexposed humans *Science* **370**, 89-94

7. Tarke, A., Coelho, C. H., Zhang, Z., Dan, J. M., Yu, E. D., Methot, N. *et al.* (2022) SARS-CoV-2 vaccination induces immunological T cell memory able to cross-recognize variants from Alpha to Omicron *Cell* **185**, 847-859 e811

8. Lu, X., Hosono, Y., Nagae, M., Ishizuka, S., Ishikawa, E., Motooka, D. *et al.* (2021) Identification of conserved SARS-CoV-2 spike epitopes that expand public cTfh clonotypes in mild COVID-19 patients *J Exp Med* **218**, e20211327

9. M.E. Emmelot, M. Vos, M.C. Boer, N.Y. Rots, J. de Wit, C. van Els, and P. Kaaijk, Omicron BA.1 Mutations in SARS-CoV-2 Spike Lead to Reduced T-Cell Response in Vaccinated and Convalescent Individuals. Viruses 14 (2022).

10. X. Fan, D. Cao, L. Kong, and X. Zhang, Cryo-EM analysis of the post-fusion structure of the SARS-CoV spike glycoprotein. Nat Commun 11 (2020) 3618.
